# Comparative analysis of gut microbiome alterations in early- and late-onset preeclampsia: A case control study

**DOI:** 10.1371/journal.pone.0348943

**Published:** 2026-06-18

**Authors:** Sofie Meijer, Luisa W. Hugerth, Mehrnaz Nouri, Lena Erlandsson, Shahram Lavasani, Stefan R. Hansson

**Affiliations:** 1 Department of Clinical Sciences Lund, Division of Obstetrics and Gynecology, Lund University, Lund, Sweden; 2 Department of Obstetrics and Gynecology, Skåne University Hospital, Lund, Sweden; 3 Department of Medical Biochemistry and Microbiology, Science for Life Laboratory, Uppsala University, Uppsala, Sweden; 4 ImmuneBiotech AB, Lund, Sweden; State Key Laboratory for Diagnosis and Treatment of Infectious Diseases, CHINA

## Abstract

Preeclampsia (PE) is a complication during pregnancy characterized by hypertension, organ damage, and systemic inflammation. Increasing evidence suggests that the gut microbiome may play a role in the pathophysiology of PE. However, previous studies on the gut microbiome have generally overlooked the distinction between subgroups of PE, although clinical manifestations may differ. Also, most studies have not used deep sequencing techniques. Therefore, this study aimed to explore further potential differences in gut dysbiosis in different PE subgroups compared to controls using shotgun metagenomics. We studied the bacterial gut microbiome using shotgun metagenomic sequencing in 37 pregnant patients in the third trimester from a Swedish cohort, separating patients according to subtype (healthy controls N = 21, late-onset PE N = 8, early-onset PE N = 8). Differential relative abundances and alpha diversity were evaluated using Wilcoxon rank sum test, and beta diversity was evaluated using PERMANOVA. Multiple linear regression was used to study associations between gut microbiome composition differences and clinical parameters. Late-onset PE and early-onset PE were both associated with significantly different beta diversity compared to controls. Differences remained significant after adjusting for age, and were not affected by gestational age, BMI or parity. Alpha diversity was lower in late-onset PE compared to controls. While no significant differences in taxonomic abundances were seen after correcting for multiple testing, several interesting leads were identified, including a higher abundance of genus *Blautia* in late-onset PE, and lower abundance of *Coprococcus catus* and unclassified *Lachnospiraceae* in early-onset PE. Functional analysis did not reveal any significant differences after false discovery rate (FDR) correction. In conclusion, our results showed subgroup-specific gut microbiome differences in PE with more pronounced associations in late-onset PE, despite limited power due to the observational design and small cohort. Accordingly, our results highlight the importance of subgroup analysis when studying PE.

## Introduction

Preeclampsia (PE) is a pregnancy-related inflammatory disease characterized by hypertension and organ damage, manifesting after 20 gestational weeks (gw). It is a leading cause of maternal and fetal morbidity and mortality worldwide [1]. The clinical manifestations and disease severity can vary greatly, and severe PE is defined as the following features: early-onset PE (onset <34 gw); blood pressure (BP) >160/110 mmHg; hemolysis, elevated liver enzymes and low platelets (HELLP) syndrome; severe organ dysfunction; and/or eclampsia [1]. The etiology is partly unknown, but the placenta is central to pathophysiology, as described in the two-stage model of PE [2]. In early-onset PE, shallow invasion of the trophoblast into the decidual spiral arterioles leads to poor placentation and malperfusion, causing oxidative- and syncytiotrophoblast stress [3]. Late-onset PE (≥34 gw) is thought to be more related to maternal risk factors, syncytiotrophoblast stress and eventually senescence of the placenta. A dysfunctional placenta results in the maternal syndrome of PE, with an increased inflammatory response and oxidative stress as part of the systemic reaction causing the general endothelial damage pathognomonic for PE [2,4]. Early-onset PE seems to be more related to an increased inflammatory response than late-onset PE, including a decrease in regulatory T (Treg) cell activity [5–8].

The gut microbiome has gained increasing attention as a possible factor in the pathophysiology of PE, as well as in many other diseases involving low-grade inflammation such as obesity, diabetes, and hypertension [9–12]. Maternal obesity is also considered a risk factor for PE. Several PE studies, mainly performed in Asian populations, have shown that PE is associated with a less favorable gut microbiome composition [13–18]. Among others, the probiotic genus *Bifidobacteria* has been shown to be depleted in PE [17]. Miao et al showed a negative correlation between *Bifidobacterium* abundance and systolic blood pressure (SBP), diastolic blood pressure (DBP), cholesterol levels, and aspartate aminotransferase (ASAT), and a highly significant negative correlation with triglyceride levels [17].

The gut microbiome diversity and composition are affected by both environmental and host factors such as diet, and ethnicity, causing a high variation in the gut microbiome between populations [19]. Using 16S rRNA sequencing, our previous study showed that PE is associated with dysbiosis of the gut microbiome in a Swedish cohort, characterized by higher levels of phylum *Bacteroidetes* and lower levels of phylae *Verrucomicrobia*, *Synergistetes*, and genera *Cloacibacillus* and *Akkermansia*. We also showed a tendency towards lower abundance of the class *Actinobacteria* in PE, a class mainly consisting of *Bifidobacteria*. In addition, a correlation between gastrointestinal symptoms and PE was shown, suggesting that the gut microbiota and gastrointestinal symptoms might be a part of the maternal syndrome [20].

Concentrations of lipopolysaccharide (LPS) have been found to be elevated in plasma and feces in PE, indicating that gut dysbiosis might lead to endotoxemia that contributes to systemic inflammation through toll-like receptor 4 (TLR4) signaling [15,21]. Increased plasma levels, of the gut microbiota-derived metabolite trimethylamine-N-oxide (TMAO), which is a strong prognostic marker for chronic kidney disease and adverse cardiac events [22–24], have also been found to be elevated in PE [15]. Gut bacteria influence their host by producing various short-chain fatty acids (SCFAs). Notably, lower levels of SCFAs, such as butyrate, along with a reduction in butyrate-producing bacterial taxa, have been observed in PE [25]. Some taxa have been correlated with PE parameters such as BP, proteinuria, oedema, and inflammatory markers such as IL-6 [7,9]. Consumption of probiotic milk during pregnancy is associated with lower risk of severe PE, including late-onset PE with severe features [26], although randomized controlled studies are lacking. The gut microbiome might be a potential target for therapeutics in PE.

Although there is increasing evidence that the gut microbiome plays a role in PE, previous studies have generally overlooked the distinction between subgroups of PE. Gut dysbiosis may exacerbate inflammation, and thereby clinical manifestations and symptoms. However, as the clinical phenotype can vary greatly between PE subgroups, we hypothesize that the role of the gut microbiome may also differ. Additionally, most studies have utilized 16S rRNA sequencing rather than deep sequencing techniques. Therefore, this study aimed to explore further potential differences in gut dysbiosis in different PE subgroups compared to controls using shotgun metagenomics.

## Materials and methods

### Ethics statement

The study was approved by the Regional Ethics Committee at Lund University, Sweden (Study number 2017/419), and conducted in accordance with the Declaration of Helsinki. All subjects gave their written informed consent before entering the study. All samples were coded before analysis.

### Study cohort and sample collection

In our previous study [20], a larger cohort of women with singleton normotensive pregnancy or newly diagnosed PE in the third trimester were recruited between 04/10/2017 and 10/02/2020 at Skåne University Hospital and antenatal care centers in Scania, Sweden. Subjects were matched according to gestational length and recruited in the same area of Scania. The 16S rRNA gene amplicon sequencing was successfully performed on a total of 15 PE (7 early-onset, 8 late-onset), and 24 controls (C) (8 before 34 gw, 16 after 34 gw). For this study, subjects were chosen based on availability of samples, where there were remaining feces stored from a subset of the original cohort (16 PE patients and 21 C), hence there is an overlap (11 PE and 19 C) with samples used for 16S sequencing in our previous study [20]. The remaining samples were analysed using shotgun metagenomics, aiming to further evaluate potential differences between subgroups of PE. The demographic data of the groups was compared to ensure the groups were comparable.

Preeclampsia was defined according to the International Society for the Study of Hypertension in Pregnancy (ISSHP) 2018 diagnostic criteria [1], i.e., *de novo* hypertension (≥ 140/90 mmHg) after gestational week 20, and proteinuria and/or signs of organ dysfunction. Early-onset PE was defined as manifesting before gw 34, and late-onset PE after gw 34. Exclusion criteria were diabetes (including gestational diabetes), celiac disease, inflammatory bowel disease, BMI > 30 before pregnancy, and antibiotic treatment within the previous 2 weeks. The BMI threshold was set due to known differences in the gut microbiota composition in obese pregnant women [27].

Demographic data for all three groups and results from routine blood sample analyses performed on PE patients (platelets, aspartate aminotransferase (ASAT), alanine aminotransferase (ALAT), creatinine, uric acid, albumin) were acquired from medical records. A questionnaire regarding diet and gastrointestinal symptoms was filled in at inclusion, and plasma and feces samples were collected and stored at −80°C. Plasma (P) and feces (F) calprotectin levels were analysed using a two-site sandwich ELISA technique (Immundiagnostik AG, Bensheim, Germany). Details regarding data and sample collection, as well as lab analyses, are described in our previous study [20].

### Shotgun metagenomic sequencing and metagenomic analyses

Fresh-frozen feces samples were sent on dry ice to CosmosID (Germantown, MD, USA) for DNA extraction and shotgun metagenomic sequencing. DNA from samples was isolated using the QIAGEN DNeasy PowerSoil Pro Kit, according to the manufacturer’s protocol. Extracted DNA samples were quantified using Qubit Flex fluorometer and Qubit™ dsDNA HS Assay Kit (Thermofisher Scientific). DNA libraries were prepared using the Nextera XT DNA Library Preparation Kit (Illumina) and Nextera Index Kit (Illumina) with total DNA input of 1 ng. Genomic DNA was fragmented using a proportional amount of Illumina Nextera XT fragmentation enzyme. Combinatory dual indexes were added to each sample followed by 12 cycles of PCR to construct libraries. DNA libraries were purified using AMpure magnetic Beads (Beckman Coulter) and eluted in QIAGEN EB buffer. DNA libraries were quantified using Qubit fluorometer and Qubit™ dsDNA HS Assay Kit. Libraries were then sequenced on an Illumina HiSeq X platform at 2x150bp.

Raw taxonomic reads were filtered with fastp [28] for the removal of adapters and low-quality bases and depleted of human DNA with kraken2 [29] using the hg19 reference human genome. The remaining reads were annotated taxonomically with Metaphlan4 [30] against the CHOCOPhlAn vOct22 database and functionally with HUMAnN3 [31]. Functional assignments were then converted to gut metabolic modules with Omixer-rpmR [32].

All further bioinformatic analyses were performed in R v.4.3.1 using the vegan package v.2.7−1. Taxa were excluded if they did not meet at least one of the following criteria: a relative abundance of 0.005% in at least 7 samples, or a relative abundance of at least 0.1% in at least 2 samples. Of 990 taxa originally detected, 348 were kept. After exclusion, a row called “other taxa” was added to the matrix to preserve compositionality after the exclusion of low abundance taxa; the median relative abundance of “other taxa” was 2%. Alpha (within-sample) diversity indices were calculated as observed species number, Shannon’s entropy and Pielou’s evenness. Beta (between-samples) diversity was based on Bray-Curtis dissimilarities. Homogeneity of dispersion was assessed with the betdisper function. The Wilcoxon rank sum test was used for significance testing in relative abundance and alpha diversity indices, and a PERMANOVA analysis with 999 permutations was performed for beta diversity significance testing. Results with *p* values < 0.05 were considered significant. Differential abundance of species, genera, metabolic and gut-brain modules was calculated in Maaslin3, considering only differential abundance, but not prevalence, and adjusting for age, sequencing depth and gut transit time, defined as slow (defecate less than 5 times per week, and never soft stools), regular, or fast (defecate at least daily, of which at least half of stools are soft). Although no features were significant after FDR, we have further explored features with a nominal p-value <0.05 for at least one PE subgroup, and where the direction of change was the same for total PE and at least one PE subgroup. These candidate features were correlated with clinical parameters with generalized linear models adjusting for the same factors as in the Maaslin3 analysis.

### Additional statistical analyses

The IBM SPSS Statistics software version 27 was used for analysis of demographic data, calprotectin values, and data collected from questionnaires and medical records. Demographic data was compared pairwise using Mann-Whitney U-test for quantitative variables, and Fisher’s exact test for categorical variables.

## Results

### Study cohort characteristics

A total of 21 C patients, and 16 PE patients with singleton pregnancies in the third trimester were included in the study. The PE patients were divided into two subgroups: late-onset PE (N = 8); and early-onset PE (N = 8), previously defined in the material and methods section. Patient demographics, selected questionnaire answers (diet, probiotic food consumption, and gastrointestinal symptoms), and PE routine blood sample analyses are summarized in Table 1. All three groups were similar in pre-pregnancy BMI, age, parity, smoking habits, diet, and probiotic food consumption, according to Mann-Whitney U-test (quantitative variables) and Fisher’s exact test (categorical variables). Gestational age at inclusion was overlapping, although the median gestational age was significantly lower in early-onset PE (median 32 weeks, 0 days) compared to controls (median 35 weeks, 4 days). Frequency of loose/watery stool was significantly higher in the early-onset PE, but not late-onset PE. Gestational age at delivery, and fetal birth weight were both significantly lower in the PE subgroups compared to C. P-calprotectin was significantly higher in both PE subgroups, while no difference was seen in F-calprotectin. No significant differences were seen in routine blood samples between the two PE subgroups, except for plasma albumin, which was significantly lower in late-onset PE compared to early-onset PE.

**Table 1:** Patient demographics, questionnaire answers, and biochemical lab results.

Parameter	C	Late-onset PE	Early-onset PE	*p* values (Late-onset PE/Early-onset PE)
N =	21	8	8	
Age (years)	31 (28 – 33.5)	32.5 (27.5 – 35)	30.5 (29 – 32.5)	0.624/1.000
Pre-pregnancy BMI (kg/m^2^)	23.3 (21.4 – 24.1)	22.9 (21.2 – 25.6)	20.9 (20.2 – 23.7)	0.919/0.234
Nulliparous, n/N (%)	16/21 (76.2 %)			0.448/0.557
Gestational age at inclusion (weeks + days)	35 + 4 (31 + 5 – 37 + 0)	36 + 4 (35 + 3 – 38 + 2)	32 + 0 (29 + 6 – 34 + 0)	0.213/0.032*
Gestational age at delivery (weeks + days)	40 + 0 (39 + 3 – 41 + 1)	37 + 2 (36 + 2 – 38 + 6)	33 + 4 (30 + 2 – 34 + 1)	0.001**/<0.001***
Birth weight (g)	3656 (3252 – 4012)	2755 (2476 – 3045)	1738 (1069 – 2071)	0.001**/<0.001***
Smoker, n/N (%):				
No	20/21 (95.2%)	7/8 (87.5%)	8/8 (100%)	
Yes	0/21 (0%)	0/8 (0%)	0/8 (0%)	
Quit before pregnancy	1/21 (4.8%)	1/8 (12.5%)	0/8 (0%)	0.685
Diet, n/N (%):				
Vegan	1/21 (4.8%)	0/8 (0%)	0/8 (0%)	
Lacto-ovo vegetarian	5/21 (23.8%)	0/8 (0%)	2/8 (25%)	
Omnivorous	15/21 (71.4%)	8/8 (100%)	6/8 (75%)	0.594
Probiotic food items consumed				
> 1 time/week (number)	1.0 (1.0 – 2.0)	1.5 (1.0 – 2.0)	1.0 (1.0 – 2.8)	0.444/0.614
Stool frequency, n/N (%):				
<3 times/week	2/21 (9.5%)	0/8 (0%)	0/8 (0%)	
3-5 times/week	8/21 (38.1%)	2/8 (25%)	2/8 (25%)	
1 time/day	9/21 (42.9%)	3/8 (37.5%)	5/8 (62.5%)	
>1 time/day	2/21 (9.5%)	3/8 (37.5%)	1/8 (12.5%)	0.102/0.277
Loose/watery stool frequency, n/N (%):				
Never/rarely	15/21 (71.4%)	3/7 (42.9%)	2/8 (25%)	
Around 25% of the time	2/21 (9.5%)	3/7 (42.9%)	3/8 (37.5%)	
Around 50% of the time	2/21 (9.5%)	0/7 (0%)	1/8 (12.5%)	
Around 75% of the time	2/21 (9.5%)	1/7 (14.3%)	2/8 (25%)	
Always, 100% of the time	0/21 (0%)	0/7 (0%)	0/8 (0%)	0.290/0.042*
SBP (mmHg)	117 (108 – 127)	145 (140 – 156)	155 (150-160)	<0.001***/<0.001***
DBP (mmHg)	70 (68 – 76)	100 (95 – 100)	103 (96 – 109)	<0.001***/<0.001***
Plasma calprotectin (µg/L)	1540 (948 – 2450)	13639 (3415 – 24342)	3109 (2442 – 4157)	<0.001***/0.004**
Feces calprotectin (mg/kg)	32 (22 – 58)	12 (3 – 26)	22 (9 – 230)	0.071/0.884
Platelets (x 10^9^/L)	-	213 (156 – 257)	196 (139 – 232)	0.563
ASAT (µkat/L)	-	0.38 (0.27 – 0.52)	0.36 (0.30 – 0.51)	0.958
ALAT (µkat/L)	-	0.28 (0.21 – 0.42)	0.35 (0.17 – 0.42)	0.753
Creatinine (µmol/L)	-	65 (56 – 69)	56 (46 – 65)	0.113
Uric acid (µmol/L)	-	347 (300 – 367)	371 (327 – 391)	0.156
Albumin (g/L)	-	24.5 (23.0 – 28.5)	30.0 (25.8 – 31.8)	0.042*

Numbers presented as medians (interquartile ranges, Q1–Q3). The Mann–Whitney U-test was used for pairwise significance testing compared with C except for smoking, and diet, where Fisher’s exact test was used. * *p* < 0.05, ** *p* < 0.01, *** *p* < 0.001. ALAT = alanine amino transferase, ASAT = aspartate amino transferase, BMI = body mass index, C = control, DBP = diastolic blood pressure, PE = preeclampsia, SBP = systolic blood pressure.

### Diversity Indices

**Fig 1:**
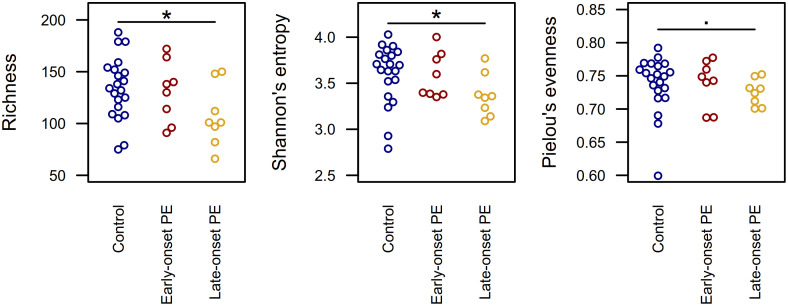
Alpha diversity in PE subgroups vs C. a) Richness, b) Shannon’s entropy, c) Pielou’s evenness.

Alpha and beta diversity analyses were performed to evaluate if there were overall gut microbiome compositional differences in PE subgroups compared with C. Late-onset PE had lower microbial diversity than C (Richness: C: 134 (IQR 116−152); Late-onset PE: 101 (IQR 93.25–121); p = 0.042; Shannon’s entropy: C: 3.39 (IQR 3.34–3.80), Late-onset PE: 3.68 (IQR 3.58–3.78); p = 0.036; Pielou’s evenness: C: 0.749 (IQR 0.728–0.765); Late-onset PE: 0.727 (IQR 0.709–0.736; p = 0.08) (Fig 1). There were no significant differences between the alpha-diversity in C compared to early-onset PE (Richness: 134 (IQR 109.5–146); Shannon”s: 3.5 (IQR 3.31–3.65); Pielou's: 0.746 (IQR: 0.727–0.763); all p > 0.6).

**Fig 2:**
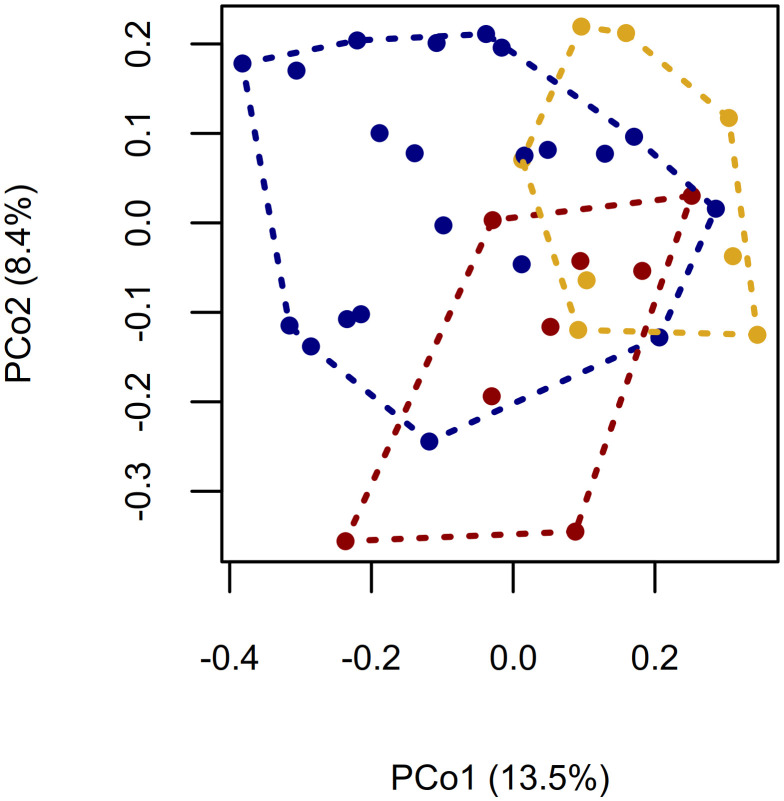
Beta diversity based on Bray-Curtis dissimilarities. The first two principal coordinates are shown. Dark blue dots: controls; yellow dots: late-onset preeclampsia; red dots: early-onset preeclampsia. Dashed lines mark the observed limit of each group.

Beta diversity was significantly different between C and subgroups of PE (adonis2: R2 = 0.091, p = 0.003; beta-disper: p = 0.09) (Fig 2). Since the low p-value of the beta-dispersion analysis suggested an effect of group size, we also assessed controls against all PE cases combined, which was also significant and had no effect of group size (adonis2: R2 = 0.060, p = 0.002; beta-disper p = 0.60). Beta diversity was correlated with the age of participants (R² = 0.041, p = 0.047), but not with parity, BMI or gestational week. Both PE and PE subgroups remained significant after adjusting for age.

### Gut microbiome taxa abundances

The gut microbiome composition was analysed in subgroups of PE compared with C, adjusting for the effects of age, gut transit time and sequencing depth, to evaluate potential differences between different phenotypes of PE. This analysis was performed at the species and genus levels, as well as for a set of 119 bacterial pathways with known metabolic or neuroactive activity. While no taxa or pathways were significant past multiple testing correction, promising leads include higher abundance of genera *Blautia* and *Eggerthella*, and species *Roseburia inulinivorans* in late-onset PE, and lower abundance of unclassified *Lachnospiraceae* family members GGB3653 and GGB3653 SGB4964, and species *Coprococcus catus*, and higher abundance of species *Eggerthella lenta* in early-onset PE. Potentially interesting functional pathways include increased glutamate synthesis II in late-onset PE, and increased pectine degradation II, serine degradation II, and butyrate synthesis I in early-onset PE. Promising leads are listed in Table 2.

**Table 2:** Summary of taxa with differential relative abundances in subgroups of PE, compared with C.

Feature	Feature type / description	C, median abundance (prevalence)	Late-onset PE, median abundance (prevalence)	Early-onset PE, median abundance (prevalence)	Control vs. Late-onset PE, p-value (q-value)	Control vs. Early-onset PE, p-value (q-value)	Control vs. All PE p-value (q-value)
GGB9760 SGB15374	Phylum *Firmicutes*	11% (60%)	0 (37.5%)	0.5% (50%)	0.12 (0.88)	**0.034 (0.71)**	**0.017 (0.66)**
*Blautia*	Family *Lachnospiraceae*	10.1% (100%)	17.1% (100%)	12.5% (100%)	**0.012 (0.71)**	0.12 (0.84)	**0.012 (0.72)**
GGB3653	Family *Lachnospiraceae*	0.25% (85%)	0 (12.5%)	0.01% (50%)	0.49 (0.86)	**0.044 (0.71)**	**0.041 (0.72)**
GGB3653 SGB4964	Family *Lachnospiraceae*	24.7% (85%)	0 (12.5%)	1.8% (50%)	0.49 (0.89)	**0.044 (0.71)**	**0.041 (0.72)**
*Coprococcus catus*	Family *Lachnospiraceae*	42.5% (85%)	39.7% (87.5%)	30.5% (100%)	0.42 (0.88)	**0.006 (0.70)**	**0.031 (0.69)**
*Roseburia inulinivorans*	Family *Lachnospiraceae*	0% (45%)	12.0% (62.5%)	20.1% (87.5%)	**0.031 (0.71)**	**0.017 (0.70)**	**0.006 (0.66)**
*Eggerthella*	Family *Eggertelaceae*	0.004% (55%)	0.11% (50%)	0.019% (50%)	**0.041 (0.71)**	0.066 (0.71)	**0.012 (0.72)**
*Eggerthella lenta*	Family *Eggertelaceae*	0.42% (55%)	11.4% (50%)	1.9% (50%)	0.066 (0.81)	**0.041 (0.73)**	**0.012 (0.66)**
MF0004	pectine degradation II	3.37 (50%)	13.20 (87.5%)	15.14 (100%)	0.08 (0.8)	**0.045 (0.7)**	**0.013 (0.76)**
MF0048	Serine degradation	43.06 (100%)	57.66 (100%)	58.31 (100%)	0.3 (0.9)	**0.039 (0.7)**	0.057 (0.81)
MGB007	Glutamate synthesis II	50.68 (100%)	69.71 (100%)	67.00 (100%)	**0.049 (0.7)**	0.29 (0.92)	0.058 (0.81)
MGB009	Histamine synthesis	0 (35%)	0 (37.5%)	0 (37.5%)	0.91 (0.98)	0.6 (0.98)	**0.011 (0.76)**
MGB022	GABA synthesis II	0.46 (60%)	1.05 (75%)	0.59 (62.5%)	0.09 (0.8)	0.057 (0.69)	**0.031 (0.76)**
MGB052	Butyrate synthesis I	2.39 (55%)	4.32 (87.5%)	9.92 (87.5%)	0.125 (0.84)	**0.001 (0.38)**	0.053 (0.76)

### Multiple linear regression analyses

**Fig 3:**
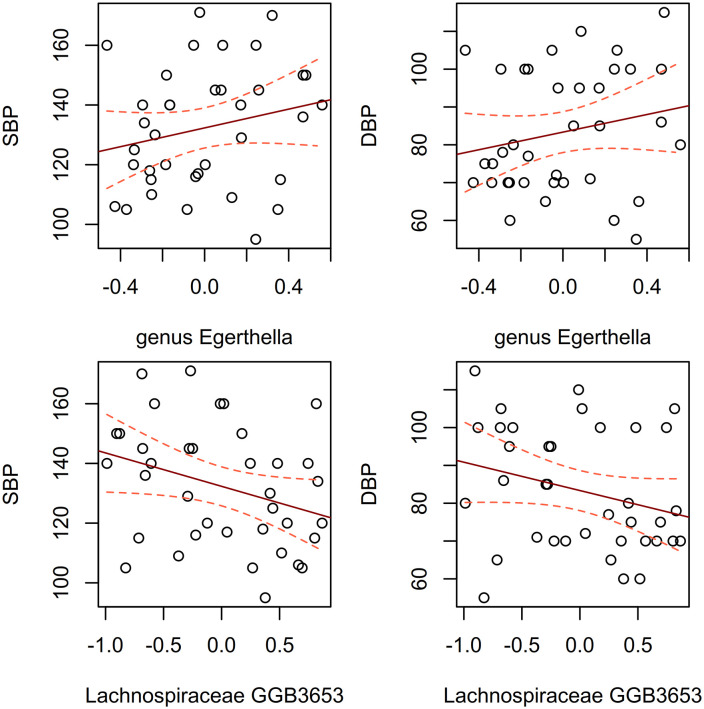
Genus *Eggerthella* has strong positive correlations with systolic blood pressure (SBP) and diastolic blood pressure (DBP), while uncultured *Lachnospiraceae* genus GGB3653 has negative correlations with both.

Multiple linear regression analyses were performed to evaluate associations between potentially interesting taxa (significantly differentially abundant before correcting for multiple testing with the false discovery rate (FDR) procedure) in any subgroup of PE, and clinical parameters relevant to PE (Table 3). The variables SBP, DBP, gestational age at birth, birth weight, P- and F-calprotectin were analysed across the entire cohort (N = 37), while routine blood samples were only available for PE patients (N = 16). All analyses were adjusted for age and BMI. Significant associations were found most often with systolic blood pressure and various taxa, while gut metabolic modules had one weak correlation each (table 3). After adjusting for multiple testing, four correlations remained, all between two genera (*Eggerthella* and the uncultured genus GGB3653), and both SBP and DBP (Table 3, Fig 3).

**Table 3:** Significant results from multiple linear regression models on clinical parameters and potentially interesting taxa and functional pathways in late-onset or early-onset PE, including values after FDR correction.

Dependent variable	Independent variable	Partial slope coefficient (SE)	p-value	After FDR
*Eggerthella*	SBP	41.4 (10.3)	**0.00028**	**0.0043**
*Eggerthella*	DBP	27.3 (8.3)	**0.0039**	**0.029**
*GGB3653*	SBP	−20.6 (5.1)	**0.00029**	**0.0043**
*GGB3653*	DBP	−13.9 (4.4)	**0.0030**	**0.029**
*Blautia*	Platelets	45.0 (19.5)	**0.036**	0.21
*Eggerthella lenta*	SBP	22.8 (10.3)	**0.034**	0.36
*Eggerthella lenta*	Albumin	−5.6 (2.5)	**0.042**	0.36
*GGB3653 SGB4964*	SBP	−26.5 (12.8)	**0.046**	0.39
*GGB9760 SGB15374*	SBP	−18.2 (8.1)	**0.032**	0.36
*GGB9760 SGB15374*	Albumin	5.6 (1.8)	**0.009**	0.36
MGB007	Uric acid	181 (76.9)	**0.034**	0.66
MF0004	Albumin	12.6 (5.5)	**0.039**	70.65
MGB052	Platelets	−1065 (363)	**0.011**	0.65

SBP = systolic blood pressure, DBP = diastolic blood pressure, FDR = false discovery rate, SE = standard error

## Discussion

In this study, we showed that patients with early-onset PE and late-onset PE display subgroup-specific differences in gut microbiome compositions when compared to C, with larger differences in late-onset PE, including a lower microbial diversity. Both late-onset PE and early-onset PE were associated with a significant difference in beta diversity compared with C. Importantly, beta diversity did not correlate with gestational age, BMI, or parity, and remained significantly different after adjusting for age. Our results suggest that PE is associated with an altered gut microbiome composition at the global level, and these alterations may play different roles in the pathophysiology of PE subgroups.

A well-known issue in high dimensional data such as metagenomics is multiple comparisons, resulting in an increased rate of false positive results. We chose to adjust for multiple comparisons using FDR correction, resulting in no taxa showing significantly differential relative abundances despite differences in alpha and beta diversity. It is possible that the sample size was too small to show significant differences in specific taxa. Although non-significant after FDR correction, interesting leads for taxa possibly involved in PE include several members of the *Lachnospiraceae* family. These leads may primarily be seen as hypothesis generating, and some are discussed further below. Further studies with larger sample sizes are needed to determine any true differences in taxa abundances. It is possible that other factors mediate microbiome differences as well, such as diet change and medications related to PE.

Late-onset PE was associated with a higher median relative abundance of family *Lachnospiraceae* member *Blautia*, compared with controls. These differences are in line with previous studies mainly performed in China [14,17,33,34], except from one study showing lower *Blautia* in PE [34], and previous studies showing lower unclassified *Lachnospiraceae* in PE [35,36].

*Blautia* is a known butyrate-producer within the family *Lachnospiraceae*, and although the genus is commonly regarded as beneficial [37], it has also been indicated in diseases such as IBS, gestational diabetes mellitus, and PE [14,33,38,39]. Miao et al showed a positive correlation in PE between *Blautia* and age, pregestational weight, C-reactive protein, triglyceride, and low-density lipoprotein-cholesterol [17]. In non-pregnant individuals, high abundance of *Blautia* is also associated with glucose metabolism disturbances, arterial hypertension, low consumption of resistant starch, and high-fat diet [40]. Low dietary fiber intake has also been associated with PE [41], and a high-fat diet has been shown to aggravate PE symptoms in a PE mouse model [42].

Our results also indicated a strong positive correlation between the genus *Eggerthella*, SBP and DBP. Higher abundance of *Eggerthella* has been reported in non-pregnant hypertensive patients previously [43], but has not been indicated in PE, to the best of our knowledge. Interestingly, our results also showed a significant negative correlation between unclassified *Lachnospiraceae* member GGB3653 and SBP as well as DBP. Previous studies have shown depleted unclassified *Lachnospiraceae* in PE [35,36], in line with our results. Members of this taxon are suggested to have a protective effect against PE [44], being among the main producers of SCFAs including butyrate [37]. In fact, lower SCFA levels have been indicated in PE [25,34,45]. However, the role of *Lachnospiraceae* is controversial, and different members of *Lachnospiraceae* have also been associated with diseases such as obesity [46]. Worth mentioning is also that the probiotic species *Coprococcus catus* was lower in early-onset PE, which has been observed in previous studies of PE as well, even before clinical onset [13,25]. Further studies are needed to correlate dysbiosis, clinical parameters and SCFAs levels in PE.

Our previous study showed that PE was associated with significantly more frequent bowel movements, and a tendency towards more loose/watery stool [20]. Our current subgroup analysis showed that early-onset, but not late-onset, PE was associated with a higher rate of loose/watery stool. It is possible that gastrointestinal manifestations differ between PE phenotypes, although further studies in larger cohorts are needed.

Although none remained significant after FDR correction, analyses of the functional potential of the gut microbiome in PE versus normal pregnancy revealed several potentially interesting pathways with known metabolic or neuroactive activity. However, very little is known about what effects these pathways might have in PE. Further studies are needed to elucidate which pathways are potentially involved in PE.

Previous studies have generally overlooked the distinction between subgroups of PE, often failing to examine them separately. As PE is a heterogeneous condition with diverse clinical manifestations, this lack of differentiation may partly account for inconsistencies in the literature. Additionally, variations in study populations may contribute to the discrepancies observed across studies. Our results indicate that both late-onset PE and early-onset PE are associated with gut dysbiosis, although possibly with larger differences in late-onset PE, as suggested by significantly lower alpha diversity. We hypothesize that late-onset PE might be more driven by a combination of host and environmental risk factors, such as gut dysbiosis, while early-onset PE is more associated with early defects in placentation. This might have important implications for interventional strategies as well. To date, low-dose acetyl salicylic acid is used as a prophylactic treatment during pregnancy in individuals with an increased risk of PE, but treatment mainly lowers the risk of early-onset disease [47]. We speculate that interventions targeting the gut microbiome, such as probiotics, might have a larger effect on late-onset PE.

A key strength of this study is the focus on distinct subgroups of PE, acknowledging that underlying mechanisms and clinical manifestations might differ between them. However, the relatively small size of these subgroups represents a limitation, reducing statistical power and potentially affecting the robustness of the findings. Future studies aimed at confirming and expanding these findings should include larger, well-characterized cohorts and clearly defined subgroups. In addition, longitudinal studies are warranted to investigate whether microbiome differences can be detected as early as in the first trimester, as suggested by previous reports [25].

## Conclusions

Our findings reveal robust differences in the gut microbiome composition in both early-onset and late-onset PE, despite limited power due to the observational design and small cohort. Late-onset PE was associated with more significant differences, characterized by a significantly lower alpha diversity in addition to differences in beta diversity. Based on our results, we hypothesize that host and environmental factors such as the gut microbiome might be more involved in the pathophysiology of late-onset PE. Collectively, our results highlight the importance of subgroup-specific analysis and consideration of population-related differences in PE research. However, validation in larger and more diverse cohorts is essential to substantiate these findings and clarify the role of the gut microbiome in the development and progression of PE.

## References

[1] Brown MA, Magee LA, Kenny LC, Karumanchi SA, McCarthy FP, Saito S, et al. The hypertensive disorders of pregnancy: ISSHP classification, diagnosis & management recommendations for international practice. Pregnancy Hypertens. 2018;13:291–310. doi: 10.1016/j.preghy.2018.05.004 29803330

[2] Redman CWG, Staff AC, Roberts JM. Syncytiotrophoblast stress in preeclampsia: the convergence point for multiple pathways. Am J Obstet Gynecol. 2022;226(2S):S907–27. doi: 10.1016/j.ajog.2020.09.047 33546842

[3] Staff AC. The two-stage placental model of preeclampsia: An update. J Reprod Immunol. 2019;134–135:1–10. doi: 10.1016/j.jri.2019.07.004 31301487

[4] Borzychowski AM, Sargent IL, Redman CWG. Inflammation and pre-eclampsia. Semin Fetal Neonatal Med. 2006;11(5):309–16. doi: 10.1016/j.siny.2006.04.001 16828580

[5] Hu W, Wang H, Wang Z, Huang H, Dong M. Elevated serum levels of interleukin-15 and interleukin-16 in preeclampsia. J Reprod Immunol. 2007;73(2):166–71. doi: 10.1016/j.jri.2006.06.005 16938352

[6] Mellembakken JR, Aukrust P, Hestdal K, Ueland T, Abyholm T, Videm V. Chemokines and leukocyte activation in the fetal circulation during preeclampsia. Hypertension. 2001;38(3):394–8. doi: 10.1161/01.hyp.38.3.394 11566911

[7] Cudihy D, Lee RV. The pathophysiology of pre-eclampsia: current clinical concepts. J Obstet Gynaecol. 2009;29(7):576–82. doi: 10.1080/01443610903061751 19757258

[8] Steinborn A, Schmitt E, Kisielewicz A, Rechenberg S, Seissler N, Mahnke K, et al. Pregnancy-associated diseases are characterized by the composition of the systemic regulatory T cell (Treg) pool with distinct subsets of Tregs. Clin Exp Immunol. 2012;167(1):84–98. doi: 10.1111/j.1365-2249.2011.04493.x 22132888 PMC3248090

[9] Cani PD, Osto M, Geurts L, Everard A. Involvement of gut microbiota in the development of low-grade inflammation and type 2 diabetes associated with obesity. Gut Microbes. 2012;3(4):279–88. doi: 10.4161/gmic.19625 22572877 PMC3463487

[10] Cani PD, Amar J, Iglesias MA, Poggi M, Knauf C, Bastelica D, et al. Metabolic endotoxemia initiates obesity and insulin resistance. Diabetes. 2007;56(7):1761–72. doi: 10.2337/db06-1491 17456850

[11] Saad MJA, Santos A, Prada PO. Linking Gut Microbiota and Inflammation to Obesity and Insulin Resistance. Physiology (Bethesda). 2016;31(4):283–93. doi: 10.1152/physiol.00041.2015 27252163

[12] Jose PA, Raj D. Gut microbiota in hypertension. Curr Opin Nephrol Hypertens. 2015;24(5):403–9. doi: 10.1097/MNH.0000000000000149 26125644 PMC4578629

[13] Liu J, Yang H, Yin Z, Jiang X, Zhong H, Qiu D, et al. Remodeling of the gut microbiota and structural shifts in Preeclampsia patients in South China. Eur J Clin Microbiol Infect Dis. 2017;36(4):713–9. doi: 10.1007/s10096-016-2853-z 27988814

[14] Lv L-J, Li S-H, Li S-C, Zhong Z-C, Duan H-L, Tian C, et al. Early-Onset Preeclampsia Is Associated With Gut Microbial Alterations in Antepartum and Postpartum Women. Front Cell Infect Microbiol. 2019;9:224. doi: 10.3389/fcimb.2019.00224 31297341 PMC6608563

[15] Wang J, Gu X, Yang J, Wei Y, Zhao Y. Gut Microbiota Dysbiosis and Increased Plasma LPS and TMAO Levels in Patients With Preeclampsia. Front Cell Infect Microbiol. 2019;9:409. doi: 10.3389/fcimb.2019.00409 31850241 PMC6901393

[16] Chen X, Li P, Liu M, Zheng H, He Y, Chen M-X, et al. Gut dysbiosis induces the development of pre-eclampsia through bacterial translocation. Gut. 2020;69(3):513–22. doi: 10.1136/gutjnl-2019-319101 31900289

[17] Miao T, Yu Y, Sun J, Ma A, Yu J, Cui M, et al. Decrease in abundance of bacteria of the genus Bifidobacterium in gut microbiota may be related to pre-eclampsia progression in women from East China. Food Nutr Res. 2021;65:10.29219/fnr.v65.5781. doi: 10.29219/fnr.v65.5781 34262418 PMC8254465

[18] Liu X, Zeng X, Li X, Xin S, Zhang F, Liu F, et al. Landscapes of gut bacterial and fecal metabolic signatures and their relationship in severe preeclampsia. J Transl Med. 2024;22(1):360. doi: 10.1186/s12967-024-05143-5 38632606 PMC11022388

[19] Gupta VK, Paul S, Dutta C. Geography, Ethnicity or Subsistence-Specific Variations in Human Microbiome Composition and Diversity. Front Microbiol. 2017;8:1162. doi: 10.3389/fmicb.2017.01162 28690602 PMC5481955

[20] Meijer S, Pasquinelli E, Renzi S, Lavasani S, Nouri M, Erlandsson L, et al. Gut micro- and mycobiota in preeclampsia: bacterial composition differences suggest role in pathophysiology. Biomolecules. 2023;13(2). 10.3390/biom13020346 PMC9953204 36830715

[21] Vogl T, Tenbrock K, Ludwig S, Leukert N, Ehrhardt C, van Zoelen MAD, et al. Mrp8 and Mrp14 are endogenous activators of Toll-like receptor 4, promoting lethal, endotoxin-induced shock. Nat Med. 2007;13(9):1042–9. doi: 10.1038/nm1638 17767165

[22] Koeth RA, Wang Z, Levison BS, Buffa JA, Org E, Sheehy BT, et al. Intestinal microbiota metabolism of L-carnitine, a nutrient in red meat, promotes atherosclerosis. Nat Med. 2013;19(5):576–85. doi: 10.1038/nm.3145 23563705 PMC3650111

[23] Tang WHW, Wang Z, Kennedy DJ, Wu Y, Buffa JA, Agatisa-Boyle B, et al. Gut microbiota-dependent trimethylamine N-oxide (TMAO) pathway contributes to both development of renal insufficiency and mortality risk in chronic kidney disease. Circ Res. 2015;116(3):448–55. doi: 10.1161/CIRCRESAHA.116.305360 25599331 PMC4312512

[24] Kim RB, Morse BL, Djurdjev O, Tang M, Muirhead N, Barrett B, et al. Advanced chronic kidney disease populations have elevated trimethylamine N-oxide levels associated with increased cardiovascular events. Kidney Int. 2016;89(5):1144–52. doi: 10.1016/j.kint.2016.01.014 27083288

[25] Altemani F, Barrett HL, Gomez-Arango L, Josh P, David McIntyre H, Callaway LK, et al. Pregnant women who develop preeclampsia have lower abundance of the butyrate-producer Coprococcus in their gut microbiota. Pregnancy Hypertens. 2021;23:211–9. doi: 10.1016/j.preghy.2021.01.002 33530034

[26] Nordqvist M, Jacobsson B, Brantsæter A-L, Myhre R, Nilsson S, Sengpiel V. Timing of probiotic milk consumption during pregnancy and effects on the incidence of preeclampsia and preterm delivery: a prospective observational cohort study in Norway. BMJ Open. 2018;8(1):e018021. doi: 10.1136/bmjopen-2017-018021 29362253 PMC5780685

[27] Mokkala K, Röytiö H, Munukka E, Pietilä S, Ekblad U, Rönnemaa T, et al. Gut Microbiota Richness and Composition and Dietary Intake of Overweight Pregnant Women Are Related to Serum Zonulin Concentration, a Marker for Intestinal Permeability. J Nutr. 2016;146(9):1694–700. doi: 10.3945/jn.116.235358 27466607

[28] Chen S, Zhou Y, Chen Y, Gu J. fastp: an ultra-fast all-in-one FASTQ preprocessor. Bioinformatics. 2018;34(17):i884–90. doi: 10.1093/bioinformatics/bty560 30423086 PMC6129281

[29] Wood DE, Lu J, Langmead B. Improved metagenomic analysis with Kraken 2. Genome Biol. 2019;20(1):257. doi: 10.1186/s13059-019-1891-0 31779668 PMC6883579

[30] Blanco-Míguez A, Beghini F, Cumbo F, McIver LJ, Thompson KN, Zolfo M, et al. Extending and improving metagenomic taxonomic profiling with uncharacterized species using MetaPhlAn 4. Nat Biotechnol. 2023;41(11):1633–44. doi: 10.1038/s41587-023-01688-w 36823356 PMC10635831

[31] Franzosa EA, McIver LJ, Rahnavard G, Thompson LR, Schirmer M, Weingart G, et al. Species-level functional profiling of metagenomes and metatranscriptomes. Nat Methods. 2018;15(11):962–8. doi: 10.1038/s41592-018-0176-y 30377376 PMC6235447

[32] Darzi Y, Falony G, Vieira-Silva S, Raes J. Towards biome-specific analysis of meta-omics data. ISME J. 2016;10(5):1025–8. doi: 10.1038/ismej.2015.188 26623543 PMC5029225

[33] Lv L-J, Li S-H, Wen J-Y, Wang G-Y, Li H, He T-W, et al. Deep metagenomic characterization of gut microbial community and function in preeclampsia. Front Cell Infect Microbiol. 2022;12:933523. doi: 10.3389/fcimb.2022.933523 36189343 PMC9515455

[34] Chang Y, Chen Y, Zhou Q, Wang C, Chen L, Di W, et al. Short-chain fatty acids accompanying changes in the gut microbiome contribute to the development of hypertension in patients with preeclampsia. Clin Sci (Lond). 2020;134(2):289–302. doi: 10.1042/CS20191253 31961431

[35] Wang J, Gao Y, Ren S, Li J, Chen S, Feng J, et al. Gut microbiota-derived trimethylamine N-Oxide: a novel target for the treatment of preeclampsia. Gut Microbes. 2024;16(1):2311888. doi: 10.1080/19490976.2024.2311888 38351748 PMC10868535

[36] Wang J, Shi Z-H, Yang J, Wei Y, Wang X-Y, Zhao Y-Y. Gut microbiota dysbiosis in preeclampsia patients in the second and third trimesters. Chin Med J (Engl). 2020;133(9):1057–65. doi: 10.1097/CM9.0000000000000734 32265423 PMC7213634

[37] Vacca M, Celano G, Calabrese FM, Portincasa P, Gobbetti M, De Angelis M. The controversial role of human gut Lachnospiraceae. Microorganisms. 2020;8(4). 10.3390/microorganisms8040573 PMC7232163 32326636

[38] Crusell MKW, Hansen TH, Nielsen T, Allin KH, Rühlemann MC, Damm P, et al. Gestational diabetes is associated with change in the gut microbiota composition in third trimester of pregnancy and postpartum. Microbiome. 2018;6(1):89. doi: 10.1186/s40168-018-0472-x 29764499 PMC5952429

[39] Rajilić-Stojanović M, Biagi E, Heilig HGHJ, Kajander K, Kekkonen RA, Tims S, et al. Global and deep molecular analysis of microbiota signatures in fecal samples from patients with irritable bowel syndrome. Gastroenterology. 2011;141(5):1792–801. doi: 10.1053/j.gastro.2011.07.043 21820992

[40] Kashtanova DA, Tkacheva ON, Doudinskaya EN, Strazhesko ID, Kotovskaya YV, Popenko AS, et al. Gut Microbiota in Patients with Different Metabolic Statuses: Moscow Study. Microorganisms. 2018;6(4):98. doi: 10.3390/microorganisms6040098 30257444 PMC6313665

[41] Qiu C, Coughlin KB, Frederick IO, Sorensen TK, Williams MA. Dietary fiber intake in early pregnancy and risk of subsequent preeclampsia. Am J Hypertens. 2008;21(8):903–9. doi: 10.1038/ajh.2008.209 18636070

[42] Zhu S, Chen S, Ge Y, Zhou F, Su K, Xu C, et al. High-fat diet induces pre-eclampsia through dampening cell-autonomous C3 in trophoblasts. Commun Biol. 2025;8(1):879. doi: 10.1038/s42003-025-08298-z 40481230 PMC12144106

[43] Yan Q, Gu Y, Li X, Yang W, Jia L, Chen C, et al. Alterations of the Gut Microbiome in Hypertension. Front Cell Infect Microbiol. 2017;7:381. doi: 10.3389/fcimb.2017.00381 28884091 PMC5573791

[44] Li P, Wang H, Guo L, Gou X, Chen G, Lin D, et al. Association between gut microbiota and preeclampsia-eclampsia: a two-sample Mendelian randomization study. BMC Med. 2022;20(1):443. doi: 10.1186/s12916-022-02657-x 36380372 PMC9667679

[45] Jin J, Gao L, Zou X, Zhang Y, Zheng Z, Zhang X, et al. Gut Dysbiosis Promotes Preeclampsia by Regulating Macrophages and Trophoblasts. Circ Res. 2022;131(6):492–506. doi: 10.1161/CIRCRESAHA.122.320771 35950704

[46] Cho I, Blaser MJ. The human microbiome: at the interface of health and disease. Nat Rev Genet. 2012;13(4):260–70. doi: 10.1038/nrg3182 22411464 PMC3418802

[47] Roberge S, Bujold E, Nicolaides KH. Aspirin for the prevention of preterm and term preeclampsia: systematic review and metaanalysis. Am J Obstet Gynecol. 2018;218(3):287-293.e1. doi: 10.1016/j.ajog.2017.11.561 29138036

