# Supplementary material for: Comparative analysis of gut microbiome alterations in early- and late-onset preeclampsia: A case control study
Source: PLoS One. 2026 Jun 18;21(6):e0348943. doi: 10.1371/journal.pone.0348943 (PMC13278574; doi:10.1371/journal.pone.0348943)
Supplement: Attachment — Submitted filename: Subgroup-specific gut microbiome _ Reviewers comments.docx [file pone.0348943.s001.docx]

| **Section** | **COMMENTS** |
| --- | --- |
| **Abstract** | The aim of your study being added to the abstract would be a plus |
| **Methodology** | Clarify the process for selecting the cohorts. You mentioned that 16 PE and 21 C were analyzed, with 11 PE and 19 C overlapping with the previous 16S study. How were the specific samples chosen for this shotgun analysis? Were they picked randomly, or were they selected based on the availability of remaining samples? |
| **Discussion** | Discussing the contradictory Lachnospiraceae findings, you could more strongly emphasize that this group is highly diverse and contains both beneficial and potentially detrimental members, which explains the varying results across studies. |

The manuscript is great, technically sound and clearly presented. The conclusions are supported by the data. The methodology, using a modern sequencing technique provides better resolution compared to the more commonly used 16S rRNA sequencing. The statistical analysis was done carefully, using non-parametric tests. The authors also applied the Bonferroni correction for multiple comparisons.

The major findings were the distinct microbial profiles linked to each subgroup. The marked decrease of Bifidobacterium adolescentis in the severe PE group is a well analysed result that fits the proposed pathophysiological model of increased inflammation in this subgroup. In contrast, the increase of Faecalibacterium prausnitzii in late-onset PE is particularly interesting, as it goes against several earlier reports. The authors interpret this as a likely protective mechanism, which is an interesting hypothesis that deserves more discussion and investigation.

The limitation of the study, which the authors acknowledged, is the small sample size within the PE subgroups. This limits the statistical power to generalize the findings. This issue is clear because the Bonferroni correction, a conservative method, made all differential taxa non-significant. While relying on the LEfSe analysis and uncorrected p-values is a reasonable strategy to find potential biological signals in pilot investigations, it would help to frame these findings as exploratory and hypothesis-generating. Presenting the study as a foundational step for future research in larger, well-characterized groups would strengthen the manuscript's impact.

To further improve this manuscript, a few clarifications could be added. First, a more comprehensive breakdown of the “severe PE” group would be useful. Specifically, stating how many of the nine patients were classified based on early onset (less than 34 weeks) versus severe features showing up later would help readers understand the group. A brief explanation of this composite grouping strategy would also be useful. Secondly, a more detailed summary in the discussion that highlights which specific findings were confirmed by shotgun sequencing and which are new would clarify the value of this deeper sequencing approach. Finally, the finding about Faecalibacterium could be expanded by looking into potential reasons for the differences with other studies, such as population-specific variations in diet, genetics, or environmental factors related to the Swedish cohort
