# Supplementary material for: Comparative analysis of gut microbiome alterations in early- and late-onset preeclampsia: A case control study
Source: PLoS One. 2026 Jun 18;21(6):e0348943. doi: 10.1371/journal.pone.0348943 (PMC13278574; doi:10.1371/journal.pone.0348943)
Supplement: Attachment — Submitted filename: Peer review report PONE-D-25-56344.docx [file pone.0348943.s002.docx]

**Peer Review Report**

***Manuscript:*** *“Subgroup-specific gut microbiome alterations in preeclampsia indicate a higher impact in severe disease: a case control study”*

# 1. Overall Recommendation

**Recommendation:** Major Revision.

The topic is timely and clinically relevant, and the subgroup approach using shotgun metagenomics is a strength. However, the small sample size, limited handling of multiple testing/high-dimensionality, and some statistical/design clarifications are needed before the conclusions can be considered robust.

# 2. Summary of the Manuscript

This Swedish third‑trimester case-control study uses shotgun metagenomic sequencing of fecal samples from 37 pregnant women (controls n=21; late‑onset PE n=7; severe PE n=9) to characterize subgroup‑specific gut microbiome differences. Wilcoxon rank‑sum tests were used for differential abundances and alpha diversity, PERMANOVA for beta diversity, LEfSe for species‑level discrimination, and multiple linear regression (adjusted for age, BMI, and subgroup) for clinical associations. The main findings are greater compositional shifts in severe PE (notably lower Bifidobacterium/B. adolescentis and higher Blautia/Lachnospiraceae) and a higher Faecalibacterium abundance in late‑onset PE.

# 3. Major Comments (Scientific/Methodological)

## 3.1 Study design and cohort characterization

• Case-control design is appropriate for exploratory microbiome profiling, but **causal language should be avoided**.

• The manuscript should clearly state whether controls were matched to cases (e.g., by gestational age at sampling, parity, diet, or recruitment site). ***Currently, comparability is asserted, but matching strategy is not described.***

• **Case definition:** Severe PE definition combines early‑onset (<34 weeks), severe‑feature late‑onset, and HELLP/eclampsia. This mixture may blur biological signals. Consider reporting (even descriptively) how many severe PE were early‑onset vs late‑onset with severe features, and provide sensitivity analyses excluding early‑onset cases or stratifying by onset if feasible.

• Exclusion criteria are reasonable (BMI>30, recent antibiotics, IBD/celiac/diabetes), but ***the rationale for BMI cutoff and whether gestational diabetes was excluded should be clarified.***

• Provide a CONSORT‑style flow or recruitment figure (numbers screened/eligible/included/excluded) to improve transparency.

## 3.2 Microbiome sequencing and bioinformatics reproducibility

• Outsourcing to CosmosID is acceptable, but the paper needs more reproducible detail: DNA extraction kit/protocol, library prep, read length, platform (e.g., NovaSeq/HiSeq), and sequencing depth range per sample.

• Taxonomic assignment relies on proprietary k‑mer methods. Please provide software version/build and database release date. If possible, include parameter settings and quality‑control steps (host read removal, trimming thresholds, contamination checks).

• A 0.005% relative‑abundance cutoff may remove rare but potentially important taxa; justify this threshold for shotgun data and indicate how many taxa were retained.

## 3.3 Statistical testing for differential abundance

• Using Wilcoxon rank‑sum tests across ~120 genera yields high false discovery risk; **the authors acknowledge this.** However, Bonferroni correction is overly conservative and led to no significant taxa, **yet the narrative still emphasizes uncorrected p<0.05 results.**

• A more standard approach would use False Discovery Rate (Benjamini–Hochberg) or compositional methods (ANCOM‑BC, ALDEx2, DESeq2 with appropriate normalization). At minimum, provide FDR‑adjusted q‑values and base conclusions on those.

• Wilcoxon rank‑sum implies two‑group testing; but the study has three groups. ***Clarify whether comparisons were pairwise*** (late vs control; severe vs control) only, and whether any global test (e.g., Kruskal–Wallis) preceded pairwise testing.

• Report effect sizes with confidence intervals for key taxa, not fold‑changes alone.

## 3.4 Beta diversity / PERMANOVA

• PERMANOVA (999 permutations) is suitable for Bray-Curtis dissimilarity. However, assumptions of homogeneity of dispersion should be tested (PERMDISP). If group dispersions differ, PERMANOVA p‑values can be misleading.

• Provide R² (variance explained) and clarify whether permutations were constrained for gestational age or other covariates.

## 3.5 LEfSe analysis

• LEfSe is useful for feature ranking but is sensitive to small samples and compositionality; it should not replace corrected differential‑abundance testing. Please report whether LEfSe used default LDA+KW+Wilcoxon steps and whether any multiple‑testing correction was applied.

• Consider validating LEfSe hits with another method or presenting them as exploratory.

## 3.6 Regression analyzes with clinical variables

• Multiple linear regression with adjustment for age, BMI, and subgroup is reasonable, but the model structure is unclear: **Were genera entered one‑at‑a‑time or simultaneously?** Table 3 suggests one‑at‑a‑time. ***Please state explicitly.***

• The phrase ***“assuming equal effects of the bacterial genera in respective group”*** is unclear; does this mean no interaction terms? If so, justify. Given subgroup heterogeneity, interactions (genus × subgroup) may be informative.

• Several dependent variables were analyzed only in PE cases (n=16). With 3 covariates + genus, power is limited and coefficients may be unstable. Please report model diagnostics more fully (residual plots, leverage, VIF, outliers) or consider simpler correlations.

• Bonferroni over 9 genera per dependent variable is okay, but please pre‑specify the family of tests and avoid highlighting non‑significant associations after correction.

## 3.7 Interpretation and strength of conclusions

• The manuscript sometimes implies protective or pathogenic roles (e.g., Faecalibacterium “protective”, Bifidobacterium “greater impact”). Given cross‑sectional sampling in 3rd trimester, conclusions should be reframed as associations/hypotheses.

• Discuss alternative explanations (reverse causation from PE‑related diet change, medications, hospitalization, or gestational age differences at sampling). Severe PE were sampled earlier (median 32 weeks vs 35–36 weeks in other groups), which itself affects microbiome composition.

• If possible, include gestational age at sampling as a covariate in differential‑abundance and PERMANOVA analyses or provide sensitivity analyses.

# 4. Minor Comments (Clarity/Reporting)

• Abstract: **The abstract is clear and well structured**. Consider adding one sentence acknowledging the exploratory nature and limited power.

• Introduction: **Strong rationale.** Add a more explicit hypothesis and clarify why late‑onset vs severe subgrouping was chosen over early‑ vs late‑onset only.

• Methods: ***Correct small error:*** Table 1 says Kruskal–Wallis for categorical variables; strictly, smoking/diet are nominal and Fisher’s exact/χ² may be more appropriate. With very small counts, Fisher’s exact is preferred.

• Results: ***Provide exact p‑values (not only thresholds) for key findings***. In Table 2, ensure formatting of IQR brackets is consistent.

• Figures: Ensure Figure 1 includes axis percent variance (already partly shown) and note any batch effects. For Figures 2–3, state whether values are log‑scaled. Increase readability of labels.

• Data availability: Good. Note that clinical data available “on request” should be justified per PLOS policy; consider depositing de‑identified clinical dataset if possible.

• Declarations: Competing interest section mentions Probi® funding but says authors have no affiliations; still, clarify whether any author received honoraria/consultancies or product involvement.

• References: ***Generally appropriate and current***. Ensure all citations are complete and formatted per PLOS ONE style. A few web/resource citations (e.g., CosmosID app) should include access date.

# 5. Language and Presentation

***Overall English is good and mostly concise.*** A few places would benefit from tightening and avoiding causal phrasing. Examples: replace “protective role” with “associated with less severe features” and “greater impact” with “more pronounced associations.” Define abbreviations at first use in the main text (e.g., ALAT/ASAT already defined later).

# 6. Suggested Additional Analyzes/Edits

• Re‑analyze differential abundance using FDR correction and/or a compositional method (ANCOM‑BC/ALDEx2). Present volcano/forest plots with q‑values.

• Adjust global and taxa‑level analyses for gestational age at sampling (and possibly delivery), and report sensitivity analyses.

• Test homogeneity of dispersion for beta diversity (PERMDISP).

• Provide subgroup breakdown of severe PE (early‑onset vs late‑onset severe features) and consider sensitivity analyses by onset.

• Consider functional/pathway profiling from shotgun data (e.g., HUMAnN or CosmosID functional outputs) to strengthen biological interpretation.

# 7. Final Decision Rationale

The manuscript addresses an important question with a modern sequencing approach and clinically meaningful subgrouping. To be publishable, the analysis must better control false discovery and account for key confounding (especially gestational age at sampling), and the conclusions should be aligned with corrected results and the observational design.

***Thank you for the great effort in contributing to maternal and child health.***
