# Supplementary material for: Comparative analysis of gut microbiome alterations in early- and late-onset preeclampsia: A case control study
Source: PLoS One. 2026 Jun 18;21(6):e0348943. doi: 10.1371/journal.pone.0348943 (PMC13278574; doi:10.1371/journal.pone.0348943)
Supplement: Attachment — Submitted filename: Response to reviewers.docx [file pone.0348943.s003.docx]

# Response to reviewers

Thank you for the opportunity to resubmit a revised version of our manuscript, originally titled “Subgroup-specific gut microbiome alterations in preeclampsia indicate a higher impact in severe disease: a case control study”, manuscript ID PONE-D-25-56344, to the journal PLOS ONE (eISSN 1932-6203). Thank you also for your thorough review and much appreciated suggestions for changes. We have thoroughly worked through and reanalyzed our data, including redefining the subgroups as suggested, and incorporated the changes in the manuscript. To improve the quality of the manuscript and the bioinformatic analyses, we cooperated with Dr Luisa Hugerth, associate senior lecturer at the Department of Medical Biochemistry and Microbiology at Uppsala University, Sweden. Dr Hugerth is added as a new author in the manuscript. Changes made are highlighted in the new draft.

In the following response to reviewers, we address the comments from the Peer Review Report, the email regarding the decision from PLOS One, and the attached Word document “Subgroup-specific gut microbiome _ Reviewers comments”.

***Peer Review Report***

***Manuscript:*** *“Subgroup-specific gut microbiome alterations in preeclampsia indicate a higher impact in severe disease: a case control study”*

# *1. Overall Recommendation*

***Recommendation:*** *Major Revision.*

*The topic is timely and clinically relevant, and the subgroup approach using shotgun metagenomics is a strength. However, the small sample size, limited handling of multiple testing/high-dimensionality, and some statistical/design clarifications are needed before the conclusions can be considered robust.*

# *2. Summary of the Manuscript*

*This Swedish third-trimester case-control study uses shotgun metagenomic sequencing of fecal samples from 37 pregnant women (controls n=21; late-onset PE n=7; severe PE n=9) to characterize subgroup-specific gut microbiome differences. Wilcoxon rank-sum tests were used for differential abundances and alpha diversity, PERMANOVA for beta diversity, LEfSe for species-level discrimination, and multiple linear regression (adjusted for age, BMI, and subgroup) for clinical associations. The main findings are greater compositional shifts in severe PE (notably lower Bifidobacterium/B. adolescentis and higher Blautia/Lachnospiraceae) and a higher Faecalibacterium abundance in late-onset PE.*

# *3. Major Comments (Scientific/Methodological)*

## *3.1 Study design and cohort characterization*

**Comment:** *Case-control design is appropriate for exploratory microbiome profiling, but causal language should be avoided.*

**Response:** Thank you for pointing this out. We have changed the language, accordingly, clarifying the exploratory nature of the manuscript.

**Comment:** *The manuscript should clearly state whether controls were matched to cases (e.g., by gestational age at sampling, parity, diet, or recruitment site). Currently, comparability is asserted, but matching strategy is not described.*

**Response:** The matching strategy has been clarified accordingly under “Study cohort and sample collection”. In our previous study, the subjects were originally matched according to recruitment site and gestational age. This study is based on remaining samples from our previous study, and here the gestational age differs between the early-onset PE group and controls. In general, early-onset PE gestational age at delivery cannot per definition be matched to a normal pregnancy that occurs after 37 gestational weeks. However, our analysis shows that the gut microbiome composition in our cohort was not affected by gestational age.

**Comment: *Case definition:*** *Severe PE definition combines early-onset (<34 weeks), severe-feature late-onset, and HELLP/eclampsia. This mixture may blur biological signals. Consider reporting (even descriptively) how many severe PE were early-onset vs late-onset with severe features, and provide sensitivity analyses excluding early-onset cases or stratifying by onset if feasible.*

**Response:** Thank you for this valuable suggestion. After thorough consideration, we have decided to redefine our subgroups based on gestational age at onset of PE only, to minimize mixture of clinical phenotypes and provide more robust results. This resulted in two groups (early- vs late-onset PE) consisting of 8 patients per group. Two cases previously included in the “severe PE” subgroup are now included in the “late-onset PE” group, and one case was moved from late-onset to early-onset PE.

**Comment:** *Exclusion criteria are reasonable (BMI>30, recent antibiotics, IBD/celiac/diabetes), but* ***the rationale for BMI cutoff and whether gestational diabetes was excluded should be clarified.***

**Response:** Thank you for pointing out the need for clarification. The BMI threshold was set due to known differences in the gut microbiota composition in obese pregnant women, as stated under the “Study cohort and sample collection” section. All types of diabetes, including gestational diabetes, were excluded. This has been clarified in the manuscript as suggested.

**Comment:** *Provide a CONSORT-style flow or recruitment figure (numbers screened/eligible/included/excluded) to improve transparency.*

**Response:** We agree that including a flowchart showing the recruitment process would have been a valuable addition to the study. However, due to the small size of the study with few investigators participating in recruitment, not all patients possibly eligible were screened for participation. Also, very few of the subjects asked to participate declined. Hence, the number included are virtually the same as the number screened.

## *3.2 Microbiome sequencing and bioinformatics reproducibility*

**Comment:** *Outsourcing to CosmosID is acceptable, but the paper needs more reproducible detail: DNA extraction kit/protocol, library prep, read length, platform (e.g., NovaSeq/HiSeq), and sequencing depth range per sample.*

**Response:** Thank you for pointing this out. We have added more details regarding DNA extraction, library preparation and sequencing to the “Shotgun metagenomic sequencing and metagenomic analyses” section under Materials and methods.

**Comment:** *Taxonomic assignment relies on proprietary k-mer methods. Please provide software version/build and database release date. If possible, include parameter settings and quality-control steps (host read removal, trimming thresholds, contamination checks).*

**Response:** In the revised manuscript, all bioinformatic analyses were revised and re-done from start, improving our methods with support from Dr. Hugerth. Quality control steps and methods for taxonomic assignment have been updated in the “Shotgun metagenomic sequencing and metagenomic analyses” section.

**Comment:** *A 0.005% relative-abundance cutoff may remove rare but potentially important taxa; justify this threshold for shotgun data and indicate how many taxa were retained.*

**Response:** Thank you for this valuable concern. We agree that setting the cutoff higher might remove important low-abundant taxa. We chose to set the cutoff at 0.005% as this was the cutoff we used in our previous study on an overlapping cohort, and as our groups are small which results in lower power.

## *3.3 Statistical testing for differential abundance*

**Comment:** *Using Wilcoxon rank-sum tests across ~120 genera yields high false discovery risk;* ***the authors acknowledge this.*** *However, Bonferroni correction is overly conservative and led to no significant taxa,* ***yet the narrative still emphasizes uncorrected p<0.05 results.*** *A more standard approach would use False Discovery Rate (Benjamini–Hochberg) or compositional methods (ANCOM-BC, ALDEx2, DESeq2 with appropriate normalization). At minimum, provide FDR-adjusted q-values and base conclusions on those.*

**Response:** Thank you for this valuable comment. We have performed extensive bioinformatic reanalyses, and revised the manuscript accordingly. Using FDR correction did not yield any significant taxa. However, we still find it valuable to discuss the uncorrected results, as this is a small study with an exploratory nature, and findings are in line with previous studies. To avoid confusion, this has been clarified in the discussion. Our significant results showing differences in beta diversity, as well as lower alpha diversity in late-onset PE, remain significant.

**Comment:** *Wilcoxon rank-sum implies two-group testing; but the study has three groups.* ***Clarify whether comparisons were pairwise*** *(late vs control; severe vs control) only, and whether any global test (e.g., Kruskal–Wallis) preceded pairwise testing.*

**Response:** Comparisons were made pairwise, comparing the two PE subgroups with controls separately. This has been clarified under “Additional statistical analyses” in the Materials and methods section.

**Comment:** *Report effect sizes with confidence intervals for key taxa, not fold-changes alone.*

**Response:** Thank you for this consideration. In the updated manuscript, interquartile ranges are reported for alpha diversity, but not specific taxa. For taxa, we opted to include the median abundance and prevalence of each taxon in the main table; however, we also provide the full Maaslin3 results for species, genera and modules in the supplement.

## *3.4 Beta diversity / PERMANOVA*

**Comment:** *PERMANOVA (999 permutations) is suitable for Bray-Curtis dissimilarity. However, assumptions of homogeneity of dispersion should be tested (PERMDISP). If group dispersions differ, PERMANOVA p-values can be misleading.*

**Response:** Thank you for pointing this out. After reanalyzing our data, we have also clarified the assessment of homogeneity of dispersion using the betdisper function in the manuscript.

**Comment:** *Provide R² (variance explained) and clarify whether permutations were constrained for gestational age or other covariates.*

**Response:** Thank you for your comment. The R^2^ values have been added, as suggested. Beta-diversity was correlated with age, but not gestational age or BMI. Both PE subgroups remained significant after adjusting for age. This information has been added to the results.

## *3.5 LEfSe analysis*

**Comment:** *LEfSe is useful for feature ranking but is sensitive to small samples and compositionality; it should not replace corrected differential-abundance testing. Please report whether LEfSe used default LDA+KW+Wilcoxon steps and whether any multiple-testing correction was applied.*

**Response:** Thank you for pointing out this concern. As we reanalyzed our data using redefined subgroups, we have left out the LEfSe analysis as it can result in false positive results, and replaced it with Maaslin3.

**Comment:** *Consider validating LEfSe hits with another method or presenting them as exploratory.*

**Response:** Thank you for this suggestion. As mentioned above, LEfSe analysis was left out in our reanalysis with redefined subgroups, as it can result in false positive results. Instead, we used Maaslin3.

## *3.6 Regression analyzes with clinical variables*

**Comment:** *Multiple linear regression with adjustment for age, BMI, and subgroup is reasonable, but the model structure is unclear:* ***Were genera entered one-at-a-time or simultaneously?*** *Table 3 suggests one-at-a-time.* ***Please state explicitly.***

**Response:** Genera were entered one at a time, while co-variables (age, sampling depth, gut transit time) are present in all models. This is now clarified in the methods.

**Comment:** *The phrase* ***“assuming equal effects of the bacterial genera in respective group”*** *is unclear; does this mean no interaction terms? If so, justify. Given subgroup heterogeneity, interactions (genus × subgroup) may be informative.*

**Response:** We agree that there could be interaction effects, even with our newly defined subgroups. However, given the limited power of this study, we have focused on identifying bacterial factors that are common to all subgroups.

**Comment:** *Several dependent variables were analyzed only in PE cases (n=16). With 3 covariates + genus, power is limited and coefficients may be unstable. Please report model diagnostics more fully (residual plots, leverage, VIF, outliers) or consider simpler correlations.*

**Response:** We have now opted for a simpler linear model.

**Comment:** *Bonferroni over 9 genera per dependent variable is okay, but please pre-specify the family of tests and avoid highlighting non-significant associations after correction.*

**Response:** We now only report the correlations between taxa and clinical parameters that are robust to FDR.

## *3.7 Interpretation and strength of conclusions*

**Comment:** *The manuscript sometimes implies protective or pathogenic roles (e.g., Faecalibacterium “protective”, Bifidobacterium “greater impact”). Given cross-sectional sampling in 3rd trimester, conclusions should be reframed as associations/hypotheses.*

**Response:** Thank you for this valuable point. We have revised the manuscript using a more nuanced language.

**Comment:** *Discuss alternative explanations (reverse causation from PE-related diet change, medications, hospitalization, or gestational age differences at sampling). Severe PE were sampled earlier (median 32 weeks vs 35–36 weeks in other groups), which itself affects microbiome composition.*

**Response:** Thank you for this suggestion. Possible alternative explanations have been added in the discussion. Although there was a significant difference in gestational age between early-onset PE and C (median gestational age 32 weeks 0 days, and 35 weeks 4 days, respectively), analysis showed that the differences in beta diversity were not affected by gestational age at inclusion.

**Comment:** *If possible, include gestational age at sampling as a covariate in differential-abundance and PERMANOVA analyses or provide sensitivity analyses.*

**Response:** Thank you for this consideration. As mentioned in previous comments, beta diversity was correlated with age, but not with gestational age, parity or BMI. In differential-abundance analysis, we adjusted for age, gut transit time and sequencing depth, but not gestational age, as our results showed no significant effect on overall composition.

# *4. Minor Comments (Clarity/Reporting)*

**Comment:** *Abstract:* ***The abstract is clear and well structured****. Consider adding one sentence acknowledging the exploratory nature and limited power.*

**Response:** Thank you for this suggestion. We have rephrased parts of the abstract according to similar comments and added a sentence acknowledging limited power.

**Comment:** *Introduction:* ***Strong rationale.*** *Add a more explicit hypothesis and clarify why late-onset vs severe subgrouping was chosen over early- vs late-onset only.*

**Response:** Thank you for these considerations. The hypothesis has been clarified in the end of the Introduction section. As suggested in the other comments, we have, after careful consideration, re-defined the groups into early-onset and late-onset PE. Please see previous response.

**Comment:** *Methods:* ***Correct small error:*** *Table 1 says Kruskal–Wallis for categorical variables; strictly, smoking/diet are nominal and Fisher’s exact/χ² may be more appropriate. With very small counts, Fisher’s exact is preferred.*

**Response:** Thank you for pointing this out. We have reperformed the analyses using Fisher’s exact text, and updated Table 1 accordingly.

**Comment:** *Results:* ***Provide exact p-values (not only thresholds) for key findings****. In Table 2, ensure formatting of IQR brackets is consistent.*

**Response:** Thank you for pointing this out. We have revised all p values and formatting to make sure everything is consistent.

**Comment:** *Figures: Ensure Figure 1 includes axis percent variance (already partly shown) and note any batch effects. For Figures 2–3, state whether values are log-scaled. Increase readability of labels.*

**Response:** As the data has been re-analysed, the figures have been updated.

**Comment:** *Data availability: Good. Note that clinical data available “on request” should be justified per PLOS policy; consider depositing de-identified clinical dataset if possible.*

**Response:** We have discussed this issue with a data steward at Lund University. As the data contains information that could indirectly identify participants, the dataset cannot be published openly. We have deposited the dataset in a repository with limited access (SND). The data and availability statement has been updated as follows, and a DOI will be provided upon publication:

“The metagenomics datasets generated and analyzed during the current study are available in the European Nucleotide Archive (ENA) at EMBL-EBI under accession number PRJEB79379 (https://www.ebi.ac.uk/ena/browser/view/PRJEB79379). The clinical data contains information that could compromise the privacy of research participants. The data is a confidential document according to Swedish law and cannot be made openly available. A description of the data and information about how to request access are available at [DOI will be added when the manuscript is accepted]”

**Comment:** *Declarations: Competing interest section mentions Probi® funding but says authors have no affiliations; still, clarify whether any author received honoraria/consultancies or product involvement.*

**Response:** Thank you for pointing this out. We have added a sentence clarifying that no honoraria/consultancies or product involvement were received.

**Comment:** *References: Generally appropriate and current. Ensure all citations are complete and formatted per PLOS ONE style. A few web/resource citations (e.g., CosmosID app) should include access date.*

**Response:** Thank you for your comment. The citations have been formatted according to PLOS ONE guidelines.

# *5. Language and Presentation*

**Comment:** *Overall English is good and mostly concise. A few places would benefit from tightening and avoiding causal phrasing. Examples: replace “protective role” with “associated with less severe features” and “greater impact” with “more pronounced associations.” Define abbreviations at first use in the main text (e.g., ALAT/ASAT already defined later).*

**Response:** Thank you for these considerations regarding phrasing. We have updated the manuscript accordingly.

# *6. Suggested Additional Analyzes/Edits*

**Comment:** *Re-analyze differential abundance using FDR correction and/or a compositional method (ANCOM-BC/ALDEx2). Present volcano/forest plots with q-values.*

**Response:** Thank you for this valuable comment. All data in the revised manuscript has been reanalysed using FDR correction where applicable.

**Comment:** *Adjust global and taxa-level analyses for gestational age at sampling (and possibly delivery), and report sensitivity analyses.*

**Response:** Thank you for this consideration. As mentioned in previous comments, beta diversity was correlated with age, but not with gestational age, parity or BMI. In differential-abundance analysis, we adjusted for age, gut transit time and sequencing depth, but not gestational age, as our results showed no significant effect on overall composition.

**Comment:** *Test homogeneity of dispersion for beta diversity (PERMDISP).*

**Response:** Thank you for pointing this out. As mentioned under previous comments, homogeneity of dispersion was assessed with the betdisper function. This information has been added to the “Shotgun metagenomic sequencing and metagenomic analyses” section.

**Comment:** *Provide subgroup breakdown of severe PE (early-onset vs late-onset severe features) and consider sensitivity analyses by onset.*

**Response:** Thank you for this valuable consideration. We have redefined the groups as suggested in the other comments – please see our response to previous comments.

**Comment:** *Consider functional/pathway profiling from shotgun data (e.g., HUMAnN or CosmosID functional outputs) to strengthen biological interpretation.*

**Response:** Thank you for this valuable point. We agree that functional profiling further strengthens the manuscript and have now added this as suggested and also related the results to clinical symptoms when possible.

# *7. Final Decision Rationale*

*The manuscript addresses an important question with a modern sequencing approach and clinically meaningful subgrouping. To be publishable, the analysis must better control false discovery and account for key confounding (especially gestational age at sampling), and the conclusions should be aligned with corrected results and the observational design.*

***Thank you for the great effort in contributing to maternal and child health.***

**Comment:** *1. Please ensure that your manuscript meets PLOS ONE's style requirements, including those for file naming. The PLOS ONE style templates can be found at*

[*https://journals.plos.org/plosone/s/file?id=wjVg/PLOSOne_formatting_sample_main_body.pdf*](https://journals.plos.org/plosone/s/file?id=wjVg/PLOSOne_formatting_sample_main_body.pdf) *and*

[*https://journals.plos.org/plosone/s/file?id=ba62/PLOSOne_formatting_sample_title_authors_affiliations.pdf*](https://journals.plos.org/plosone/s/file?id=ba62/PLOSOne_formatting_sample_title_authors_affiliations.pdf)

**Response:** Thank you for pointing this out. We have now revised the manuscript in accordance with PLOS ONE’s style requirements.

**Comment:** *2. Thank you for stating the following in the Acknowledgments Section of your manuscript:*

*“This project was funded by the Swedish Research Council (grant number 314373.35.135949), ALF, Region Skåne, the SUS Foundations, and Probi®.”*

*We note that you have provided funding information that is not currently declared in your Funding Statement. However, funding information should not appear in the Acknowledgments section or other areas of your manuscript. We will only publish funding information present in the Funding Statement section of the online submission form.*

*Please remove any funding-related text from the manuscript and let us know how you would like to update your Funding Statement. Currently, your Funding Statement reads as follows:*

*“This project was funded by the Swedish Research Council (grant number 314373.35.135949, S.R.H), ALF (S.R.H), Region Skåne (S.R.H), the SUS Foundations (S.R.H), and Probi® (S.R.H). Funders did not play any role in the study design, data collection and analysis, decision to publish, or preparation of the manuscript. Funder URLs:* [*https://www.vr.se/english.html*](https://www.vr.se/english.html)*;* [*https://vard.skane.se/en/skane-university-hospital/research-and-education/research2/*](https://vard.skane.se/en/skane-university-hospital/research-and-education/research2/)*;* [*https://www.probi.se/en*](https://www.probi.se/en)*”*

*Please include your amended statements within your cover letter; we will change the online submission form on your behalf.*

**Response:** Thank you for noting this. The funding information has been removed from the manuscript. We have further added funding information for Dr. Hugerth. The new statement reads as follows:

“This project was funded by the Swedish Research Council (grant number 314373.35.135949, S.R.H), ALF (S.R.H), Region Skåne (S.R.H), the SUS Foundations (S.R.H), and Probi® (S.R.H). L.W.H is supported by the SciLifeLab & Wallenberg Data Driven Life Science Program (grant: KAW 2020.0239). Funders did not play any role in the study design, data collection and analysis, decision to publish, or preparation of the manuscript. Funder URLs: <https://www.vr.se/english.html>; <https://vard.skane.se/en/skane-university-hospital/research-and-education/research2/>; <https://www.probi.se/en>; <https://www.scilifelab.se/data-driven/>”

**Comment:** *3. We note that the grant information you provided in the ‘Funding Information’ and ‘Financial Disclosure’ sections do not match.*

*When you resubmit, please ensure that you provide the correct grant numbers for the awards you received for your study in the ‘Funding Information’ section.*

**Response:** Thank you for noting this. The previous grant numbers are accurate. We have further added funding information for Dr. Hugerth.

**Comment:** *4. In the online submission form you indicate that your data is not available for proprietary reasons and have provided a contact point for accessing this data. Please note that your current contact point is a co-author on this manuscript. According to our Data Policy, the contact point must not be an author on the manuscript and must be an institutional contact, ideally not an individual. Please revise your data statement to a non-author institutional point of contact, such as a data access or ethics committee, and send this to us via return email. Please also include contact information for the third party organization, and please include the full citation of where the data can be found.*

**Response:** We have discussed this issue further with a data steward at Lund University. Please, see our response and updated data and availability statement under previous similar comment.

**Comment:** *5. Your ethics statement should only appear in the Methods section of your manuscript. If your ethics statement is written in any section besides the Methods, please delete it from any other section.*

**Response:** Thank you for pointing this out. We have now removed our ethics statements from other sections than the methods section.

**Comment:** *6. If the reviewer comments include a recommendation to cite specific previously published works, please review and evaluate these publications to determine whether they are relevant and should be cited. There is no requirement to cite these works unless the editor has indicated otherwise.*

**Response:** Thank you, this has been considered during the revision process.

*Review Comments to the Author*

**Comment:** *Reviewer #1: This is a well-written and thoughtfully structured manuscript that makes a valuable contribution to the field. The study is clearly motivated, the objectives are well-defined, and the methodology is appropriate and rigorously applied. The results are presented in a logical and coherent manner, and the discussion effectively connects the findings to existing literature while highlighting their broader implications.

The manuscript demonstrates a strong command of the subject matter and provides new insights that can be beneficial for both researchers and practitioners. The writing style is clear, concise, and engaging, making the paper accessible to a wide academic audience. Figures and tables are relevant and well-designed, supporting the narrative effectively.

Overall, this is a high-quality piece of research that meets the journal’s standards for originality, methodological soundness, and clarity. I recommend publication with only minor editorial adjustments (if any) to improve flow and readability.

No concerns were identified regarding research ethics, data integrity, or potential duplication. The study appears to have been conducted responsibly and reported transparently.*

**Response:** Thank you for recognizing the importance of our manuscript. During the revision process, we have strived to keep the same style and readability of the manuscript.

**Comment:** *Reviewer #2: The manuscript is technically sound and clearly presented. The conclusions are supported by the data. The methodology, using a modern sequencing technique provides better resolution compared to the more commonly used 16S rRNA sequencing. The statistical analysis was done carefully, using non-parametric tests. The authors also applied the Bonferroni correction for multiple comparisons.
The major findings were the distinct microbial profiles linked to each subgroup. The marked decrease of Bifidobacterium adolescentis in the severe PE group is a well analysed result that fits the proposed pathophysiological model of increased inflammation in this subgroup. In contrast, the increase of Faecalibacterium prausnitzii in late-onset PE is particularly interesting, as it goes against several earlier reports. The authors interpret this as a likely protective mechanism, which is an interesting hypothesis that deserves more discussion and investigation.
The limitation of the study, which the authors acknowledged, is the small sample size within the PE subgroups. This limits the statistical power to generalize the findings. This issue is clear because the Bonferroni correction, a conservative method, made all differential taxa non-significant. While relying on the LEfSe analysis and uncorrected p-values is a reasonable strategy to find potential biological signals in pilot investigations, it would help to frame these findings as exploratory and hypothesis-generating. Presenting the study as a foundational step for future research in larger, well-characterized groups would strengthen the manuscript's impact.
To further improve this manuscript, a few clarifications could be added. First, a more comprehensive breakdown of the “severe PE” group would be useful. Specifically, stating how many of the nine patients were classified based on early onset (less than 34 weeks) versus severe features showing up later would help readers understand the group. A brief explanation of this composite grouping strategy would also be useful. Secondly, a more detailed summary in the discussion that highlights which specific findings were confirmed by shotgun sequencing and which are new would clarify the value of this deeper sequencing approach. Finally, the finding about Faecalibacterium could be expanded by looking into potential reasons for the differences with other studies, such as population-specific variations in diet, genetics, or environmental factors related to the Swedish cohort.*

**Response:** Thank you for your valuable considerations. As we have now redefined our subgroups as suggested in previous comments into early-onset and late-onset PE, rather than mixing early-onset PE with late-onset PE with severe features, our results have naturally changed. We believe that this provides more stringent subgroups, removing the risk of subjectivity in subgrouping. This, in combination with strengthening the bioinformatic analysis with the help of Dr. Hugerth, will provide us with more stringent results.

**Comment:** *Reviewer #3: Thank you for the opportunity to review this manuscript. The study addresses an important and timely question-whether gut microbiome alterations differ by preeclampsia subgroup and the use of shotgun metagenomics is a notable strength. The subgroup focus (late-onset vs severe PE) is clinically meaningful, and the manuscript is generally well written and logically structured.

However, I recommend major revision because several methodological and statistical issues limit the strength of the inferences as currently presented. Most importantly, the sample size is small, especially within subgroups, and the “severe PE” category is heterogeneous (mixing early-onset cases and late-onset cases with severe features), which may blur biological signals. In addition, gestational age at sampling differs between groups, particularly with severe PE sampled earlier in pregnancy, and this is a well-known driver of microbiome variation; it needs to be adjusted for or explored via sensitivity analyses.

Regarding statistics, while the general choice of methods (non-parametric tests for taxa/diversity and PERMANOVA for beta diversity) is appropriate, the analysis is not sufficiently rigorous for high-dimensional microbiome data. Many highlighted taxa rely on uncorrected p-values, whereas Bonferroni correction yields no significant taxa; this mismatch should be resolved by re-analysis using FDR correction (Benjamini–Hochberg) and/or compositional differential-abundance approaches (e.g., ANCOM-BC, ALDEx2). PERMANOVA results should be accompanied by dispersion testing (PERMDISP) and reporting of explained variance (R²). Regression analyses linking genera to clinical variables are potentially interesting but are based on very small PE case numbers and require clearer model specification, diagnostics, and more cautious interpretation.

The discussion is thoughtful, but several statements drift toward causal or protective/pathogenic language that is not supported by a cross-sectional third-trimester case–control design. I recommend reframing these as associations/hypotheses and expanding consideration of reverse causation (dietary changes, medication exposure, or disease-related physiological shifts).

Data sharing is partly compliant: sequencing reads are publicly available, but the underlying clinical dataset is only “on request.” PLOS policy typically expects either a de-identified clinical dataset in a repository or a clearly defined formal restricted-access mechanism with justification. Please address this.

I did not identify concerns about dual publication or major publication-ethics issues. Ethics approval and informed consent appear appropriate, but tightening the data-availability pathway would further strengthen compliance.

In summary, this work is promising and potentially publishable, but the authors should (1) strengthen multiple-testing control and compositional analysis, (2) address gestational-age confounding and subgroup heterogeneity, (3) add missing beta-diversity diagnostics, (4) clarify and temper regression interpretation, and (5) align conclusions strictly with corrected results and observational limitations.*

**Response:** Thank you for your important considerations. As mentioned in previous responses, we have redefined the groups into early-onset PE and late-onset PE after considering your comments. We have also strengthened the bioinformatic analyses by collaborating with Dr. Hugerth, an expert in the field now added as a co-author. These changes have provided more robust results. The language has also been adjusted to avoid causal language.

Regarding data sharing, the clinical dataset has been uploaded to a repository. Please, see response to previous comments. *Details are attached in MS Word (below)*

**Comment:**

| ***Section*** | ***COMMENTS*** |
| --- | --- |
| ***Abstract*** | *The aim of your study being added to the abstract would be a plus* |
| ***Methodology*** | *Clarify the process for selecting the cohorts. You mentioned that 16 PE and 21 C were analyzed, with 11 PE and 19 C overlapping with the previous 16S study. How were the specific samples chosen for this shotgun analysis? Were they picked randomly, or were they selected based on the availability of remaining samples?* |
| ***Discussion*** | *Discussing the contradictory Lachnospiraceae findings, you could more strongly emphasize that this group is highly diverse and contains both beneficial and potentially detrimental members, which explains the varying results across studies.* |

**Response:** Thank you for your considerations. We have added the aim of our study to the abstract, and clarified that samples were chosen based on availability of remaining samples. The groups have also been redefined as mentioned in previous responses, and the Discussion section was updated accordingly.
