# Supplementary material for: Comparative analysis of gut microbiome alterations in early- and late-onset preeclampsia: A case control study
Source: PLoS One. 2026 Jun 18;21(6):e0348943. doi: 10.1371/journal.pone.0348943 (PMC13278574; doi:10.1371/journal.pone.0348943)
Supplement: Peer review history

# Peer review: Decision Letter 0

**DOI:** 10.1371/journal.pone.0348943.r001

**Authors:** Zongxin Ling

26 Nov 2025

PONE-D-25-56344

Subgroup-specific gut microbiome alterations in preeclampsia indicate a higher impact in severe disease: a case control study

PLOS ONE

Dear Dr. Meijer,

Thank you for submitting your manuscript to PLOS ONE. After careful consideration, we feel that it has merit but does not fully meet PLOS ONE’s publication criteria as it currently stands. Therefore, we invite you to submit a revised version of the manuscript that addresses the points raised during the review process.

Please respond to the reviewers' comments point by point.

Please submit your revised manuscript by Jan 10 2026 11:59PM. If you will need more time than this to complete your revisions, please reply to this message or contact the journal office at plosone@plos.org. When you're ready to submit your revision, log on to https://www.editorialmanager.com/pone/ and select the 'Submissions Needing Revision' folder to locate your manuscript file.

Please include the following items when submitting your revised manuscript:

- A rebuttal letter that responds to each point raised by the academic editor and reviewer(s). You should upload this letter as a separate file labeled 'Response to Reviewers'.
- A marked-up copy of your manuscript that highlights changes made to the original version. You should upload this as a separate file labeled 'Revised Manuscript with Track Changes'.
- An unmarked version of your revised paper without tracked changes. You should upload this as a separate file labeled 'Manuscript'.

If you would like to make changes to your financial disclosure, please include your updated statement in your cover letter. Guidelines for resubmitting your figure files are available below the reviewer comments at the end of this letter.

If applicable, we recommend that you deposit your laboratory protocols in protocols.io to enhance the reproducibility of your results. Protocols.io assigns your protocol its own identifier (DOI) so that it can be cited independently in the future. For instructions see: https://journals.plos.org/plosone/s/submission-guidelines#loc-laboratory-protocols. Additionally, PLOS ONE offers an option for publishing peer-reviewed Lab Protocol articles, which describe protocols hosted on protocols.io. Read more information on sharing protocols at https://plos.org/protocols?utm_medium=editorial-email&utm_source=authorletters&utm_campaign=protocols.

We look forward to receiving your revised manuscript.

Kind regards,

Zongxin Ling

Academic Editor

PLOS ONE

Journal Requirements:

When submitting your revision, we need you to address these additional requirements.

1. Please ensure that your manuscript meets PLOS ONE's style requirements, including those for file naming. The PLOS ONE style templates can be found at

https://journals.plos.org/plosone/s/file?id=wjVg/PLOSOne_formatting_sample_main_body.pdf and

https://journals.plos.org/plosone/s/file?id=ba62/PLOSOne_formatting_sample_title_authors_affiliations.pdf

2. Thank you for stating the following in the Acknowledgments Section of your manuscript:

“This project was funded by the Swedish Research Council (grant number 314373.35.135949), ALF, Region Skåne, the SUS Foundations, and Probi®.”

We note that you have provided funding information that is not currently declared in your Funding Statement. However, funding information should not appear in the Acknowledgments section or other areas of your manuscript. We will only publish funding information present in the Funding Statement section of the online submission form.

Please remove any funding-related text from the manuscript and let us know how you would like to update your Funding Statement. Currently, your Funding Statement reads as follows:

“This project was funded by the Swedish Research Council (grant number 314373.35.135949, S.R.H), ALF (S.R.H), Region Skåne (S.R.H), the SUS Foundations (S.R.H), and Probi® (S.R.H). Funders did not play any role in the study design, data collection and analysis, decision to publish, or preparation of the manuscript. Funder URLs: https://www.vr.se/english.html; https://vard.skane.se/en/skane-university-hospital/research-and-education/research2/; https://www.probi.se/en”

Please include your amended statements within your cover letter; we will change the online submission form on your behalf.

3. We note that the grant information you provided in the ‘Funding Information’ and ‘Financial Disclosure’ sections do not match.

When you resubmit, please ensure that you provide the correct grant numbers for the awards you received for your study in the ‘Funding Information’ section.

4. In the online submission form you indicate that your data is not available for proprietary reasons and have provided a contact point for accessing this data. Please note that your current contact point is a co-author on this manuscript. According to our Data Policy, the contact point must not be an author on the manuscript and must be an institutional contact, ideally not an individual. Please revise your data statement to a non-author institutional point of contact, such as a data access or ethics committee, and send this to us via return email. Please also include contact information for the third party organization, and please include the full citation of where the data can be found.

5. Your ethics statement should only appear in the Methods section of your manuscript. If your ethics statement is written in any section besides the Methods, please delete it from any other section.

6. If the reviewer comments include a recommendation to cite specific previously published works, please review and evaluate these publications to determine whether they are relevant and should be cited. There is no requirement to cite these works unless the editor has indicated otherwise.

[Note: HTML markup is below. Please do not edit.]

Reviewers' comments:

Reviewer's Responses to Questions

**Comments to the Author**

1. Is the manuscript technically sound, and do the data support the conclusions?

The manuscript must describe a technically sound piece of scientific research with data that supports the conclusions. Experiments must have been conducted rigorously, with appropriate controls, replication, and sample sizes. The conclusions must be drawn appropriately based on the data presented.

Reviewer #1: Yes

Reviewer #2: Yes

Reviewer #3: Partly

**********

2. Has the statistical analysis been performed appropriately and rigorously?

Reviewer #1: Yes

Reviewer #2: Yes

Reviewer #3: No

**********

3. Have the authors made all data underlying the findings in their manuscript fully available?

The PLOS Data policy requires authors to make all data underlying the findings described in their manuscript fully available without restriction, with rare exception (please refer to the Data Availability Statement in the manuscript PDF file). The data should be provided as part of the manuscript or its supporting information, or deposited to a public repository. For example, in addition to summary statistics, the data points behind means, medians and variance measures should be available. If there are restrictions on publicly sharing data—e.g. participant privacy or use of data from a third party—those must be specified.

Reviewer #1: Yes

Reviewer #2: Yes

Reviewer #3: Yes

**********

4. Is the manuscript presented in an intelligible fashion and written in standard English?

PLOS ONE does not copyedit accepted manuscripts, so the language in submitted articles must be clear, correct, and unambiguous. Any typographical or grammatical errors should be corrected at revision, so please note any specific errors here.

Reviewer #1: Yes

Reviewer #2: Yes

Reviewer #3: Yes

**********

5. Review Comments to the Author

Please use the space provided to explain your answers to the questions above. You may also include additional comments for the author, including concerns about dual publication, research ethics, or publication ethics. (Please upload your review as an attachment if it exceeds 20,000 characters)

Reviewer #1: This is a well-written and thoughtfully structured manuscript that makes a valuable contribution to the field. The study is clearly motivated, the objectives are well-defined, and the methodology is appropriate and rigorously applied. The results are presented in a logical and coherent manner, and the discussion effectively connects the findings to existing literature while highlighting their broader implications.

The manuscript demonstrates a strong command of the subject matter and provides new insights that can be beneficial for both researchers and practitioners. The writing style is clear, concise, and engaging, making the paper accessible to a wide academic audience. Figures and tables are relevant and well-designed, supporting the narrative effectively.

Overall, this is a high-quality piece of research that meets the journal’s standards for originality, methodological soundness, and clarity. I recommend publication with only minor editorial adjustments (if any) to improve flow and readability.

No concerns were identified regarding research ethics, data integrity, or potential duplication. The study appears to have been conducted responsibly and reported transparently.

Reviewer #2: The manuscript is technically sound and clearly presented. The conclusions are supported by the data. The methodology, using a modern sequencing technique provides better resolution compared to the more commonly used 16S rRNA sequencing. The statistical analysis was done carefully, using non-parametric tests. The authors also applied the Bonferroni correction for multiple comparisons.

The major findings were the distinct microbial profiles linked to each subgroup. The marked decrease of Bifidobacterium adolescentis in the severe PE group is a well analysed result that fits the proposed pathophysiological model of increased inflammation in this subgroup. In contrast, the increase of Faecalibacterium prausnitzii in late-onset PE is particularly interesting, as it goes against several earlier reports. The authors interpret this as a likely protective mechanism, which is an interesting hypothesis that deserves more discussion and investigation.

The limitation of the study, which the authors acknowledged, is the small sample size within the PE subgroups. This limits the statistical power to generalize the findings. This issue is clear because the Bonferroni correction, a conservative method, made all differential taxa non-significant. While relying on the LEfSe analysis and uncorrected p-values is a reasonable strategy to find potential biological signals in pilot investigations, it would help to frame these findings as exploratory and hypothesis-generating. Presenting the study as a foundational step for future research in larger, well-characterized groups would strengthen the manuscript's impact.

To further improve this manuscript, a few clarifications could be added. First, a more comprehensive breakdown of the “severe PE” group would be useful. Specifically, stating how many of the nine patients were classified based on early onset (less than 34 weeks) versus severe features showing up later would help readers understand the group. A brief explanation of this composite grouping strategy would also be useful. Secondly, a more detailed summary in the discussion that highlights which specific findings were confirmed by shotgun sequencing and which are new would clarify the value of this deeper sequencing approach. Finally, the finding about Faecalibacterium could be expanded by looking into potential reasons for the differences with other studies, such as population-specific variations in diet, genetics, or environmental factors related to the Swedish cohort.

Reviewer #3: Thank you for the opportunity to review this manuscript. The study addresses an important and timely question-whether gut microbiome alterations differ by preeclampsia subgroup and the use of shotgun metagenomics is a notable strength. The subgroup focus (late-onset vs severe PE) is clinically meaningful and the manuscript is generally well written and logically structured.

However, I recommend major revision because several methodological and statistical issues limit the strength of the inferences as currently presented. Most importantly, the sample size is small, especially within subgroups, and the “severe PE” category is heterogeneous (mixing early-onset cases and late-onset cases with severe features), which may blur biological signals. In addition, gestational age at sampling differs between groups, particularly with severe PE sampled earlier in pregnancy, and this is a well-known driver of microbiome variation; it needs to be adjusted for or explored via sensitivity analyses.

Regarding statistics, while the general choice of methods (non-parametric tests for taxa/diversity and PERMANOVA for beta diversity) is appropriate, the analysis is not sufficiently rigorous for high-dimensional microbiome data. Many highlighted taxa rely on uncorrected p-values, whereas Bonferroni correction yields no significant taxa; this mismatch should be resolved by re-analysis using FDR correction (Benjamini–Hochberg) and/or compositional differential-abundance approaches (e.g., ANCOM-BC, ALDEx2). PERMANOVA results should be accompanied by dispersion testing (PERMDISP) and reporting of explained variance (R²). Regression analyses linking genera to clinical variables are potentially interesting but are based on very small PE case numbers and require clearer model specification, diagnostics, and more cautious interpretation.

The discussion is thoughtful, but several statements drift toward causal or protective/pathogenic language that is not supported by a cross-sectional third-trimester case–control design. I recommend reframing these as associations/hypotheses and expanding consideration of reverse causation (dietary changes, medication exposure, or disease-related physiological shifts).

Data sharing is partly compliant: sequencing reads are publicly available, but the underlying clinical dataset is only “on request.” PLOS policy typically expects either a de-identified clinical dataset in a repository or a clearly defined formal restricted-access mechanism with justification. Please address this.

I did not identify concerns about dual publication or major publication-ethics issues. Ethics approval and informed consent appear appropriate, but tightening the data-availability pathway would further strengthen compliance.

In summary, this work is promising and potentially publishable, but the authors should (1) strengthen multiple-testing control and compositional analysis, (2) address gestational-age confounding and subgroup heterogeneity, (3) add missing beta-diversity diagnostics, (4) clarify and temper regression interpretation, and (5) align conclusions strictly with corrected results and observational limitations.

Details are attached in MS Word

**********

6. PLOS authors have the option to publish the peer review history of their article (what does this mean?). If published, this will include your full peer review and any attached files.

If you choose “no”, your identity will remain anonymous but your review may still be made public.

**Do you want your identity to be public for this peer review?**   For information about this choice, including consent withdrawal, please see our Privacy Policy.

Reviewer #1: No

Reviewer #2: **Yes:**   Obed Woani Konutse

Reviewer #3: No

**********

[NOTE: If reviewer comments were submitted as an attachment file, they will be attached to this email and accessible via the submission site. Please log into your account, locate the manuscript record, and check for the action link "View Attachments". If this link does not appear, there are no attachment files.]

To ensure your figures meet our technical requirements, please review our figure guidelines: https://journals.plos.org/plosone/s/figures

You may also use PLOS’s free figure tool, NAAS, to help you prepare publication quality figures: https://journals.plos.org/plosone/s/figures#loc-tools-for-figure-preparation.

NAAS will assess whether your figures meet our technical requirements by comparing each figure against our figure specifications.

Attachment Submitted filename: Subgroup-specific gut microbiome _ Reviewers comments.docx

Attachment Submitted filename: Peer review report PONE-D-25-56344.docx

# Author comment: Author response to Decision Letter 1

**DOI:** 10.1371/journal.pone.0348943.r002

20 Apr 2026

The following responses have been copied from the attached "Response to reviewers" letter:

Response to reviewers

Thank you for the opportunity to resubmit a revised version of our manuscript, originally titled “Subgroup-specific gut microbiome alterations in preeclampsia indicate a higher impact in severe disease: a case control study”, manuscript ID PONE-D-25-56344, to the journal PLOS ONE (eISSN 1932-6203). Thank you also for your thorough review and much appreciated suggestions for changes. We have thoroughly worked through and reanalyzed our data, including redefining the subgroups as suggested, and incorporated the changes in the manuscript. To improve the quality of the manuscript and the bioinformatic analyses, we cooperated with Dr Luisa Hugerth, associate senior lecturer at the Department of Medical Biochemistry and Microbiology at Uppsala University, Sweden. Dr Hugerth is added as a new author in the manuscript. Changes made are highlighted in the new draft.

In the following response to reviewers, we address the comments from the Peer Review Report, the email regarding the decision from PLOS One, and the attached Word document “Subgroup-specific gut microbiome _ Reviewers comments”.

Peer Review Report

Manuscript: “Subgroup-specific gut microbiome alterations in preeclampsia indicate a higher impact in severe disease: a case control study”

1. Overall Recommendation

Recommendation: Major Revision.

The topic is timely and clinically relevant, and the subgroup approach using shotgun metagenomics is a strength. However, the small sample size, limited handling of multiple testing/high-dimensionality, and some statistical/design clarifications are needed before the conclusions can be considered robust.

2. Summary of the Manuscript

This Swedish third-trimester case-control study uses shotgun metagenomic sequencing of fecal samples from 37 pregnant women (controls n=21; late-onset PE n=7; severe PE n=9) to characterize subgroup-specific gut microbiome differences. Wilcoxon rank-sum tests were used for differential abundances and alpha diversity, PERMANOVA for beta diversity, LEfSe for species-level discrimination, and multiple linear regression (adjusted for age, BMI, and subgroup) for clinical associations. The main findings are greater compositional shifts in severe PE (notably lower Bifidobacterium/B. adolescentis and higher Blautia/Lachnospiraceae) and a higher Faecalibacterium abundance in late-onset PE.

3. Major Comments (Scientific/Methodological)

3.1 Study design and cohort characterization

Comment: Case-control design is appropriate for exploratory microbiome profiling, but causal language should be avoided.

Response: Thank you for pointing this out. We have changed the language, accordingly, clarifying the exploratory nature of the manuscript.

Comment: The manuscript should clearly state whether controls were matched to cases (e.g., by gestational age at sampling, parity, diet, or recruitment site). Currently, comparability is asserted, but matching strategy is not described.

Response: The matching strategy has been clarified accordingly under “Study cohort and sample collection”. In our previous study, the subjects were originally matched according to recruitment site and gestational age. This study is based on remaining samples from our previous study, and here the gestational age differs between the early-onset PE group and controls. In general, early-onset PE gestational age at delivery cannot per definition be matched to a normal pregnancy that occurs after 37 gestational weeks. However, our analysis shows that the gut microbiome composition in our cohort was not affected by gestational age.

Comment: Case definition: Severe PE definition combines early-onset (<34 weeks), severe-feature late-onset, and HELLP/eclampsia. This mixture may blur biological signals. Consider reporting (even descriptively) how many severe PE were early-onset vs late-onset with severe features, and provide sensitivity analyses excluding early-onset cases or stratifying by onset if feasible.

Response: Thank you for this valuable suggestion. After thorough consideration, we have decided to redefine our subgroups based on gestational age at onset of PE only, to minimize mixture of clinical phenotypes and provide more robust results. This resulted in two groups (early- vs late-onset PE) consisting of 8 patients per group. Two cases previously included in the “severe PE” subgroup are now included in the “late-onset PE” group, and one case was moved from late-onset to early-onset PE.

Comment: Exclusion criteria are reasonable (BMI>30, recent antibiotics, IBD/celiac/diabetes), but the rationale for BMI cutoff and whether gestational diabetes was excluded should be clarified.

Response: Thank you for pointing out the need for clarification. The BMI threshold was set due to known differences in the gut microbiota composition in obese pregnant women, as stated under the “Study cohort and sample collection” section. All types of diabetes, including gestational diabetes, were excluded. This has been clarified in the manuscript as suggested.

Comment: Provide a CONSORT-style flow or recruitment figure (numbers screened/eligible/included/excluded) to improve transparency.

Response: We agree that including a flowchart showing the recruitment process would have been a valuable addition to the study. However, due to the small size of the study with few investigators participating in recruitment, not all patients possibly eligible were screened for participation. Also, very few of the subjects asked to participate declined. Hence, the number included are virtually the same as the number screened.

3.2 Microbiome sequencing and bioinformatics reproducibility

Comment: Outsourcing to CosmosID is acceptable, but the paper needs more reproducible detail: DNA extraction kit/protocol, library prep, read length, platform (e.g., NovaSeq/HiSeq), and sequencing depth range per sample.

Response: Thank you for pointing this out. We have added more details regarding DNA extraction, library preparation and sequencing to the “Shotgun metagenomic sequencing and metagenomic analyses” section under Materials and methods.

Comment: Taxonomic assignment relies on proprietary k-mer methods. Please provide software version/build and database release date. If possible, include parameter settings and quality-control steps (host read removal, trimming thresholds, contamination checks).

Response: In the revised manuscript, all bioinformatic analyses were revised and re-done from start, improving our methods with support from Dr. Hugerth. Quality control steps and methods for taxonomic assignment have been updated in the “Shotgun metagenomic sequencing and metagenomic analyses” section.

Comment: A 0.005% relative-abundance cutoff may remove rare but potentially important taxa; justify this threshold for shotgun data and indicate how many taxa were retained.

Response: Thank you for this valuable concern. We agree that setting the cutoff higher might remove important low-abundant taxa. We chose to set the cutoff at 0.005% as this was the cutoff we used in our previous study on an overlapping cohort, and as our groups are small which results in lower power.

3.3 Statistical testing for differential abundance

Comment: Using Wilcoxon rank-sum tests across ~120 genera yields high false discovery risk; the authors acknowledge this. However, Bonferroni correction is overly conservative and led to no significant taxa, yet the narrative still emphasizes uncorrected p<0.05 results. A more standard approach would use False Discovery Rate (Benjamini–Hochberg) or compositional methods (ANCOM-BC, ALDEx2, DESeq2 with appropriate normalization). At minimum, provide FDR-adjusted q-values and base conclusions on those.

Response: Thank you for this valuable comment. We have performed extensive bioinformatic reanalyses, and revised the manuscript accordingly. Using FDR correction did not yield any significant taxa. However, we still find it valuable to discuss the uncorrected results, as this is a small study with an exploratory nature, and findings are in line with previous studies. To avoid confusion, this has been clarified in the discussion. Our significant results showing differences in beta diversity, as well as lower alpha diversity in late-onset PE, remain significant.

Comment: Wilcoxon rank-sum implies two-group testing; but the study has three groups. Clarify whether comparisons were pairwise (late vs control; severe vs control) only, and whether any global test (e.g., Kruskal–Wallis) preceded pairwise testing.

Response: Comparisons were made pairwise, comparing the two PE subgroups with controls separately. This has been clarified under “Additional statistical analyses” in the Materials and methods section.

Comment: Report effect sizes with confidence intervals for key taxa, not fold-changes alone.

Response: Thank you for this consideration. In the updated manuscript, interquartile ranges are reported for alpha diversity, but not specific taxa. For taxa, we opted to include the median abundance and prevalence of each taxon in the main table; however, we also provide the full Maaslin3 results for species, genera and modules in the supplement.

3.4 Beta diversity / PERMANOVA

Comment: PERMANOVA (999 permutations) is suitable for Bray-Curtis dissimilarity. However, assumptions of homogeneity of dispersion should be tested (PERMDISP). If group dispersions differ, PERMANOVA p-values can be misleading.

Response: Thank you for pointing this out. After reanalyzing our data, we have also clarified the assessment of homogeneity of dispersion using the betdisper function in the manuscript.

Comment: Provide R² (variance explained) and clarify whether permutations were constrained for gestational age or other covariates.

Response: Thank you for your comment. The R2 values have been added, as suggested. Beta-diversity was correlated with age, but not gestational age or BMI. Both PE subgroups remained significant after adjusting for age. This information has been added to the results.

3.5 LEfSe analysis

Comment: LEfSe is useful for feature ranking but is sensitive to small samples and compositionality; it should not replace corrected differential-abundance testing. Please report whether LEfSe used default LDA+KW+Wilcoxon steps and whether any multiple-testing correction was applied.

Response: Thank you for pointing out this concern. As we reanalyzed our data using redefined subgroups, we have left out the LEfSe analysis as it can result in false positive results, and replaced it with Maaslin3.

Comment: Consider validating LEfSe hits with another method or presenting them as exploratory.

Response: Thank you for this suggestion. As mentioned above, LEfSe analysis was left out in our reanalysis with redefined subgroups, as it can result in false positive results. Instead, we used Maaslin3.

3.6 Regression analyzes with clinical variables

Comment: Multiple linear regression with adjustment for age, BMI, and subgroup is reasonable, but the model structure is unclear: Were genera entered one-at-a-time or simultaneously? Table 3 suggests one-at-a-time. Please state explicitly.

Response: Genera were entered one at a time, while co-variables (age, sampling depth, gut transit time) are present in all models. This is now clarified in the methods.

Comment: The phrase “assuming equal effects of the bacterial genera in respective group” is unclear; does this mean no interaction terms? If so, justify. Given subgroup heterogeneity, interactions (genus × subgroup) may be informative.

Response: We agree that there could be interaction effects, even with our newly defined subgroups. However, given the limited power of this study, we have focused on identifying bacterial factors that are common to all subgroups.

Comment: Several dependent variables were analyzed only in PE cases (n=16). With 3 covariates + genus, power is limited and coefficients may be unstable. Please report model diagnostics more fully (residual plots, leverage, VIF, outliers) or consider simpler correlations.

Response: We have now opted for a simpler linear model.

Comment: Bonferroni over 9 genera per dependent variable is okay, but please pre-specify the family of tests and avoid highlighting non-significant associations after correction.

Response: We now only report the correlations between taxa and clinical parameters that are robust to FDR.

3.7 Interpretation and strength of conclusions

Comment: The manuscript sometimes implies protective or pathogenic roles (e.g., Faecalibacterium “protective”, Bifidobacterium “greater impact”). Given cross-sectional sampling in 3rd trimester, conclusions should be reframed as associations/hypotheses.

Response: Thank you for this valuable point. We have revised the manuscript using a more nuanced language.

Comment: Discuss alternative explanations (reverse causation from PE-related diet change, medications, hospitalization, or gestational age differences at sampling). Severe PE were sampled earlier (median 32 weeks vs 35–36 weeks in other groups), which itself affects microbiome composition.

Response: Thank you for this suggestion. Possible alternative explanations have been added in the discussion. Although there was a significant difference in gestational age between early-onset PE and C (median gestational age 32 weeks 0 days, and 35 weeks 4 days, respectively), analysis showed that the differences in beta diversity were not affected by gestational age at inclusion.

Comment: If possible, include gestational age at sampling as a covariate in differential-abundance and PERMANOVA analyses or provide sensitivity analyses.

Response: Thank you for this consideration. As mentioned in previous comments, beta diversity was correlated with age, but not with gestational age, parity or BMI. In differential-abundance analysis, we adjusted for age, gut transit time and sequencing depth, but not gestational age, as our results showed no significant effect on overall composition.

4. Minor Comments (Clarity/Reporting)

Comment: Abstract: The abstract is clear and well structured. Consider adding one sentence acknowledging the exploratory nature and limited power.

Response: Thank you for this suggestion. We have rephrased parts of the abstract according to similar comments and added a sentence acknowledging limited power.

Comment: Introduction: Strong rationale. Add a more explicit hypothesis and clarify why late-onset vs severe subgrouping was chosen over early- vs late-onset only.

Response: Thank you for these considerations. The hypothesis has been clarified in the end of the Introduction section. As suggested in the other comments, we have, after careful consideration, re-defined the groups into early-onset and late-onset PE. Please see previous response.

Comment: Methods: Correct small error: Table 1 says Kruskal–Wallis for categorical variables; strictly, smoking/diet are nominal and Fisher’s exact/χ² may be more appropriate. With very small counts, Fisher’s exact is preferred.

Response: Thank you for pointing this out. We have reperformed the analyses using Fisher’s exact text, and updated Table 1 accordingly.

Comment: Results: Provide exact p-values (not only thresholds) for key findings. In Table 2, ensure formatting of IQR brackets is consistent.

Response: Thank you for pointing this out. We have revised all p values and formatting to make sure everything is consistent.

Comment: Figures: Ensure Figure 1 includes axis percent variance (already partly shown) and note any batch effects. For Figures 2–3, state whether values are log-scaled. Increase readability of labels.

Response: As the data has been re-analysed, the figures have been updated.

Comment: Data availability: Good. Note that clinical data available “on request” should be justified per PLOS policy; consider depositing de-identified clinical dataset if possible.

Response: We have discussed this issue with a data steward at Lund University. As the data contains information that could indirectly identify participants, the dataset cannot be published openly. We have deposited the dataset in a repository with limited access (SND). The data and availability statement has been updated as follows, and a DOI will be provided upon public

Attachment Submitted filename: Response to reviewers.docx

# Editor report: Decision Letter 1

**DOI:** 10.1371/journal.pone.0348943.r003

**Authors:** Zongxin Ling, Zongxin Ling

23 Apr 2026

Comparative analysis of gut microbiome alterations in early- and late-onset preeclampsia: a case control study

PONE-D-25-56344R1

Dear Dr. Meijer,

We’re pleased to inform you that your manuscript has been judged scientifically suitable for publication and will be formally accepted for publication once it meets all outstanding technical requirements.

Within one week, you’ll receive an e-mail detailing the required amendments. When these have been addressed, you’ll receive a formal acceptance letter and your manuscript will be scheduled for publication.

An invoice will be generated when your article is formally accepted. Please note, if your institution has a publishing partnership with PLOS and your article meets the relevant criteria, all or part of your publication costs will be covered. Please make sure your user information is up-to-date by logging into Editorial Manager at Editorial Manager® and clicking the ‘Update My Information' link at the top of the page. For questions related to billing, please contact billing support.

If your institution or institutions have a press office, please notify them about your upcoming paper to help maximize its impact. If they’ll be preparing press materials, please inform our press team as soon as possible -- no later than 48 hours after receiving the formal acceptance. Your manuscript will remain under strict press embargo until 2 pm Eastern Time on the date of publication. For more information, please contact onepress@plos.org.

Kind regards,

Zongxin Ling

Academic Editor

PLOS One

Additional Editor Comments (optional):

Reviewers' comments:

# Editor report: Acceptance letter

**DOI:** 10.1371/journal.pone.0348943.r004

**Authors:** Zongxin Ling, Zongxin Ling

PONE-D-25-56344R1

PLOS One

Dear Dr. Meijer,

I'm pleased to inform you that your manuscript has been deemed suitable for publication in PLOS One. Congratulations! Your manuscript is now being handed over to our production team.

At this stage, our production department will prepare your paper for publication. This includes ensuring the following:

* All references, tables, and figures are properly cited

* All relevant supporting information is included in the manuscript submission,

* There are no issues that prevent the paper from being properly typeset

You will receive further instructions from the production team, including instructions on how to review your proof when it is ready. Please keep in mind that we are working through a large volume of accepted articles, so please give us a few days to review your paper and let you know the next and final steps.

Lastly, if your institution or institutions have a press office, please let them know about your upcoming paper now to help maximize its impact. If they'll be preparing press materials, please inform our press team within the next 48 hours. Your manuscript will remain under strict press embargo until 2 pm Eastern Time on the date of publication. For more information, please contact onepress@plos.org.

You will receive an invoice from PLOS for your publication fee after your manuscript has reached the completed accept phase. If you receive an email requesting payment before acceptance or for any other service, this may be a phishing scheme. Learn how to identify phishing emails and protect your accounts at https://explore.plos.org/phishing.

If we can help with anything else, please email us at customercare@plos.org.

Thank you for submitting your work to PLOS ONE and supporting open access.

Kind regards,

PLOS ONE Editorial Office Staff

on behalf of

Dr. Zongxin Ling

Academic Editor

PLOS One
